# The group A *Streptococcus* pathogenicity island RD2: virulence role and barriers to conjugative transfer

**DOI:** 10.1128/iai.00273-24

**Published:** 2024-11-27

**Authors:** Roshika Roshika, Sushila Baral, Ira Jain, Ashna Prabhu, Ameya Singh, Paul Sumby

**Affiliations:** 1 Department of Microbiology & Immunology, Reno School of Medicine, University of Nevada 12290 , Reno, Nevada, USA; University of Illinois Chicago, Chicago, Illinois, USA

**Keywords:** *Streptococcus pyogenes*, pathogenicity islands, conjugation, virulence, pathogenesis, molecular genetics

## Abstract

Serotype M28 isolates of the bacterial pathogen the group A *Streptococcus* (GAS; *Streptococcus pyogenes*), but not isolates of other serotypes, have a nonrandom association with cases of puerperal sepsis, a life-threatening infection that can occur in women following childbirth. In prior studies, we established that RD2, a pathogenicity island present in all M28 GAS isolates but mostly absent from other serotypes, is a factor in the M28–puerperal sepsis association. Here, we identified a significant reduction in the RD2 conjugation frequency in inter-serotype conjugation assays relative to intra-serotype assays. As isolates of most GAS serotypes produce an antiphagocytic hyaluronic acid capsule, while M28 isolates do not, we tested whether the capsule served as a barrier to RD2 acquisition or maintenance. The data showed that capsule production had no impact on the RD2 conjugation frequency or on the ability of RD2 to enhance vaginal colonization by GAS, but did inhibit the ability of RD2 to enhance GAS adherence to vaginal epithelial cell lines. Further molecular explanations for the inter-serotype barrier to RD2 conjugative transfer were investigated, and a conserved, chromosomally encoded Type I restriction–modification system was identified as being key. We also identified that RD2 modifies the GAS transcriptome, including mRNAs encoding virulence factors with adherence and dissemination roles, following exposure to human plasma. Our data provide insights into factors that contribute to the restriction of the RD2 pathogenicity island to discrete subsets of the GAS population.

## INTRODUCTION

The group A *Streptococcus* (GAS; *Streptococcus pyogenes*) is a bacterial pathogen that causes an extensive range of human diseases, from self-limiting pharyngitis and impetigo to invasive necrotizing fasciitis and puerperal sepsis (1). Puerperal sepsis is a life-threatening disease that can develop from a puerperal infection, an infection that originates in the reproductive tract of women ante- or postpartum and causes morbidity in ∼10% of new mothers globally (2). All isolates are thought to have the potential to cause each of the disease manifestations associated with GAS. However, layered over this perceived basal level of disease potential are serotype-specific differences that can enhance or reduce the frequency and/or severity of specific diseases. For example, serotype M1 and M3 isolates are associated with cases of severe invasive disease (3–6), serotype M18 isolates are associated with cases of the post-GAS infection sequela acute rheumatic fever (7), and serotype M28 isolates are associated with cases of puerperal sepsis (8–10). The molecular bases behind these GAS–serotype disease–phenotype associations have yet to be fully defined but are thought to include varying levels of altered gene content and gene expression (11–17).

**Fig 1:**
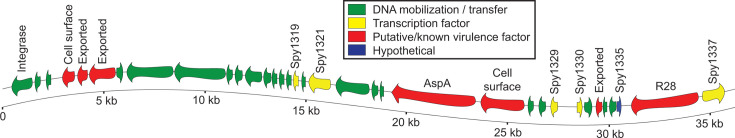
Schematic of the 36-kb RD2 pathogenicity island. Genes are represented by arrows and are color-coded based on the putative function of the encoded protein.

Common GAS typing methods include M-typing (also known as *emm*-typing), T-typing, and multilocus sequence typing (MLST) (18). M-typing divides GAS isolates into serotypes based upon the sequence of the 5’ end of the *emm* (M protein-encoding) gene, and more than 220 GAS M types have been characterized. We previously investigated the molecular basis behind the association of serotype M28 GAS isolates with cases of puerperal sepsis, identifying that the 36-kb “region of difference 2” (RD2; Fig. 1) pathogenicity island plays an important role (10, 13). The distribution of RD2 within the GAS population is restricted mostly along serotype-specific lines, with all serotype M28, and some serotype M2 and M77, isolates harboring it (9). Of note, serotype M2 GAS strains are associated with cases of vaginitis and urinary tract infections (4, 9). There are an average of eight copies of RD2 per cell, some of which are chromosomally integrated, while others are extra-chromosomal (19). Based upon sequence similarities (9), RD2 appears to have entered the GAS population from group B streptococci (GBS), which is a valuable observation, given that GBS are a normal part of the vaginal microflora in approximately 25% of women (20–22).

Conjugation represents a common mechanism for the horizontal gene transfer of genomic islands, and RD2 is no exception (23–25). In the laboratory, RD2 has been conjugated from a donor M28 GAS strain to recipient serotype M1 and M4 strains (19). Factors specifically influencing the conjugation frequency of RD2 have yet to be investigated, but more broadly, the conjugation rate is often influenced by factors such as bacterial growth phase, cell density, and mating time (26). Strain-specific characteristics of the donor and/or recipient can also influence the conjugation frequency, including physical barriers such as the presence of a capsule (27), and post-transfer barriers such as the activity of restriction–modification systems and CRISPR–Cas systems (28, 29). The GAS hyaluronic acid capsule is an important anti-phagocytic virulence factor (30). However, while isolates of most GAS serotypes are encapsulated (e.g., M1), a select few serotypes are acapsular (e.g., M28) (31, 32). The influence of the GAS capsule on RD2 conjugation has not been investigated.

Phenotypic changes induced by the presence of RD2, identified via loss-of-function studies in M28 GAS and gain-of-function studies in serotype M1, M49, and M59 isolates, include an enhanced ability to colonize the murine vagina, among other phenotypes (10, 13). Given the ability of RD2 to modify GAS disease potential, it is unclear why this element is not more widely distributed within the GAS population. In this study, we investigated potential molecular explanations for the restricted distribution of RD2 in the GAS population. We identified that the hyaluronic acid capsule, at least at the level expressed in our tested strains, does not alter the conjugation frequency of RD2 for either the donor or recipient nor does it interfere with the RD2-mediated enhancement of vaginal colonization. Rather than the capsule, we identified that serotype-specific variability in the recognition sequence of a Type I restriction–modification system serves as a major barrier to the spread of this pathogenicity island across GAS serotypes. The data further our understanding of the phenotypic consequences of RD2 acquisition and of the factors that impact its distribution.

## RESULTS

### Intra-serotype conjugation of RD2 occurs at a frequency orders of magnitude higher than that of inter-serotype conjugation

**Fig 2:**
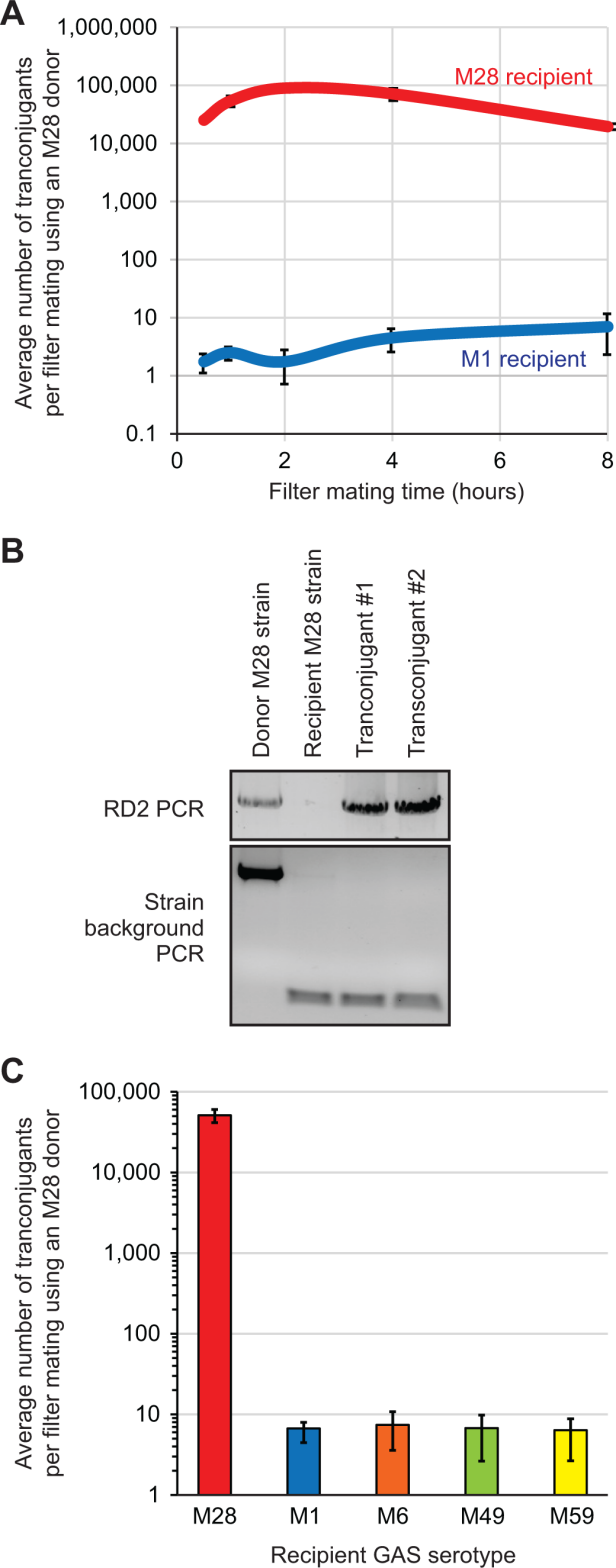
Intra-serotype conjugation of RD2 occurs at a higher frequency than inter-serotype conjugation. (**A**) RD2 conjugation data showing that the number of transconjugants differs significantly depending on whether the recipient strain is the same (M28; strain M28^KAN^ΔRD2) or a different (M1; strain M1^KAN^) serotype to the donor (M28; strain M28.RD2^SPEC2^). The influence of filter mating time also differs depending on the recipient serotype. Shown is the mean (± standard error of the mean) from four experiments. The M28 to M28 (red) and M28 to M1 (blue) data sets are statistically significantly different (ANOVA, *P* < 0.0001). For the M28 to M28 data set, analysis of variance (ANOVA) followed by Tukey’s multiple comparisons test identified significance (*P* < 0.05) between certain mating times (0.5 hours vs 2 hours, 0.5 hours vs 4 hours, 2 hours vs 8 hours, and 4 hours vs 8 hours). (**B**) Example of PCR data generated to check that putative transconjugants are indeed the recipient strain containing RD2. The presence or absence of RD2 (which is present in the donor and transconjugant strains but is absent in the recipient strain; primers UNR805/806), and of a transposon adjacent to RD2 (which is present in the donor strain but absent in the remaining strains; primers UNR286/306), was PCR-confirmed. (**C**) RD2 conjugation data further highlighting the presence of serotype-specific barriers to RD2 conjugation. The donor strain used was M28.RD2^SPEC2^, while the recipient strains were M28^KAN^ΔRD2 (M28), M1^KAN^ (M1), M6^KAN^ (M6), M49^KAN^ (M49), and M59^KAN^ (M59). Note that the data gained with the M28 recipient strain were statistically significantly different to each other strain data via ANOVA followed by Tukey’s multiple comparisons test (*P* < 0.0001), while no other pairwise comparisons were significant.

To gain insights into why the RD2 pathogenicity island is restricted to serotype M28 isolates, and a subset of M2 and M77 isolates, we tested whether there was a serotype-specific barrier to RD2 conjugation. The donor GAS strain in these assays was M28.RD2^SPEC2^, a derivative of the clinical M28 isolate MGAS6180, in which RD2 has been modified to contain a spectinomycin resistance gene. The recipient strains in these assays were M1^KAN^, which naturally lacks RD2 and is a kanamycin-resistant derivative of the clinical M1 isolate MGAS2221, and M28^KAN^ΔRD2, which is an RD2 deletion mutant derivative of MGAS6180 that is also tagged with a kanamycin resistance gene. Five filter mating times were used to enable investigation of this parameter on the number of transconjugants gained, with transconjugants being recovered by plating bacteria onto THY agar plates containing spectinomycin and kanamycin. Transconjugants arose at ~10,000 fold higher abundance in the intra-serotype conjugation (M28 to M28, red line in Fig. 2A) relative to the inter-serotype conjugation (M28 to M1, blue line in Fig. 2A) assays. That the colonies gained were the correct genetic constructs was confirmed by polymerase chain reactions (PCRs) targeting the presence/absence of RD2 and also the strain genetic background (see Fig. 2B as examples). The data are consistent, with there being a serotype-specific barrier to RD2 conjugation. In addition, filter mating times were shown to modestly impact the conjugation frequency, with statistically significant values for the intra-serotype data over time, but not for the inter-serotype data (Fig. 2A).

To assess whether the observed serotype-specific barrier to RD2-mediated conjugative transfer was applicable to a range of GAS serotypes, and not solely M1 isolates, we repeated our inter-serotype conjugation assays using serotype M6, M49, and M59 strains as recipients. Similar to the M1 data, recipient M6, M49, and M59 strains also returned only low numbers of transconjugants compared to a recipient M28 strain (Fig. 2C). Thus, the barrier to RD2-mediated transfer appears to be a common phenomenon across GAS serotypes.

### The acapsular phenotype of M28 isolates can be reversed by allelic replacement of the *hasAB* genes

**Fig 3:**
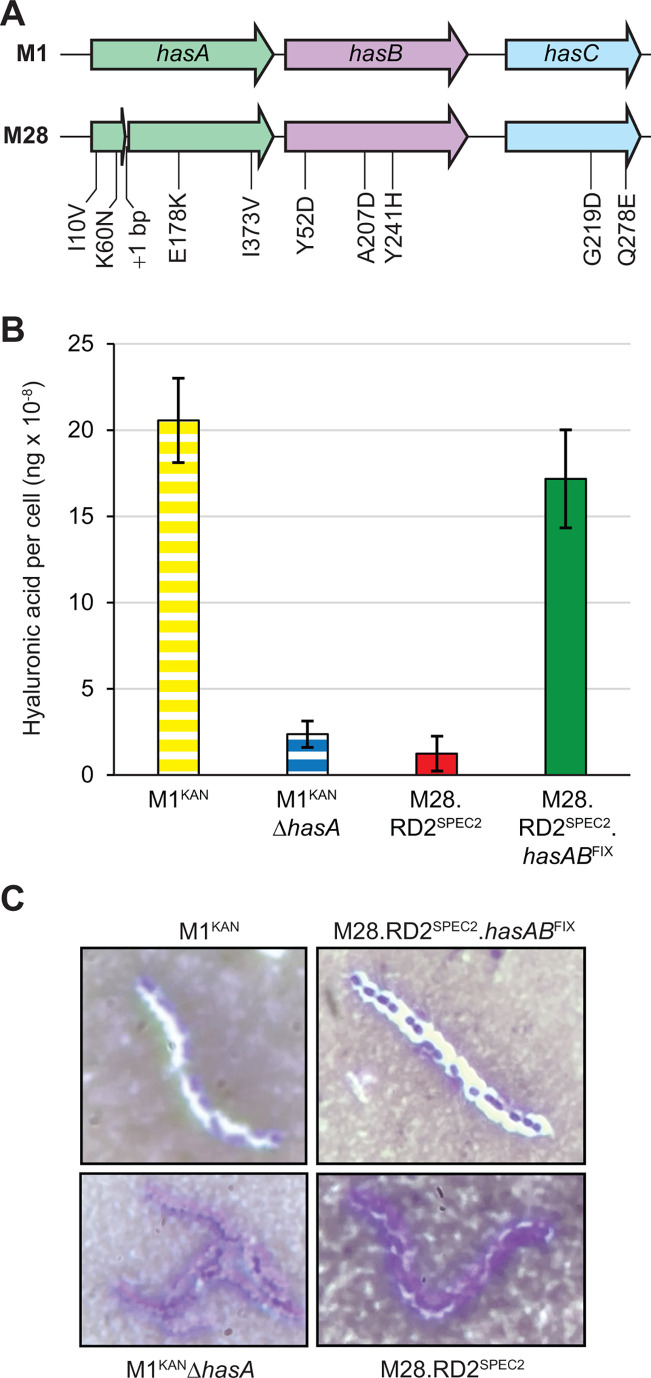
Allelic replacement of *hasAB* restores capsule expression to serotype M28 GAS. (**A**) Schematic comparing *has* operons between M1 and M28 GAS isolates. Numbering of the locations of amino acid differences between M1 and M28 GAS proteins is based upon the M1 protein sequences. (**B**) Hyaluronic acid assays. Exponential-phase cultures of the indicated GAS strains were analyzed for levels of hyaluronic acid. The experiment was performed on three occasions, using duplicate cultures of each strain in each experiment, and the values shown are the means ± standard deviations (error bars). Note that all pairwise strain comparisons are statistically significant (*P* < 0.0002), except for M1^KAN^ -vs- M28.RD2^SPEC2^.hasAB^FIX^ and of M1^KAN^ΔhasA -vs- M28.RD2^SPEC2^ (as determined by one-way ANOVA, followed by Tukey’s multiple comparisons test). (**C**) Representative India ink stain images showing the presence of a capsule in the M28 derivative strain harboring functional *hasAB* genes (strain M28.RD2^SPEC2^.hasAB^FIX^), but not in the M28 derivative harboring native M28 *hasAB* alleles (strain M28.RD2^SPEC2^).

M1, M6, M49, and M59 GAS isolates are capsular, while M28 isolates are acapsular (33). This led to the hypothesis that the difference in the intra- and inter-serotype conjugation frequency seen in Fig. 2C is due to the capsule serving as a barrier to RD2 conjugation. To facilitate testing this hypothesis, we set out to create an acapsular M1 derivative strain and a capsule-producing M28 derivative strain. Strain M1^KAN^Δ*hasA* was created by replacing the capsule biosynthesis gene *hasA* with a nonpolar erythromycin resistance cassette and, relative to its parental strain M1^KAN^, was shown to have dramatically reduced hyaluronic acid production (Fig. 3B).

As highlighted in Fig. 3A, the M28 capsule biosynthesis genes (*hasABC*) harbor ten protein-altering genetic changes relative to what is common for serotype M1 alleles (a 1-bp insertion and nine nonsynonymous SNPs), each of which could account for the acapsular phenotype. To create a capsule-producing M28 strain, we used an allelic replacement-based approach, setting out to replace the M28 *has* alleles with M1 equivalents. The first strain we created replaced the 1-bp insertion, E178K, and I373V differences in *hasA* and the Y52D difference in *hasB* (Fig. 3A), but these changes were not sufficient for capsule expression (data not shown). In our second attempt, we created an M28 derivative, M28.RD2^SPEC2^.*hasAB*^FIX^, in which we replaced all eight of the genetic alterations within *hasA* and *hasB* with their M1 counterparts. Strain M28.RD2^SPEC2^.*hasAB*^FIX^ produced hyaluronic acid in similar abundance to that seen for an M1 GAS isolate (Fig. 3B). That the increased hyaluronic acid produced by strain M28.RD2^SPEC2^.*hasAB*^FIX^ resulted in the formation of a capsule surrounding the cells was confirmed by India ink staining (Fig. 3C). Thus, we were successful in creating a capsule-producing M28 strain (M28.RD2^SPEC2^.*hasAB*^FIX^) and an acapsular M1 strain (M1^KAN^Δ*hasA*).

### Capsule does not influence the RD2 conjugation frequency

**Fig 4:**
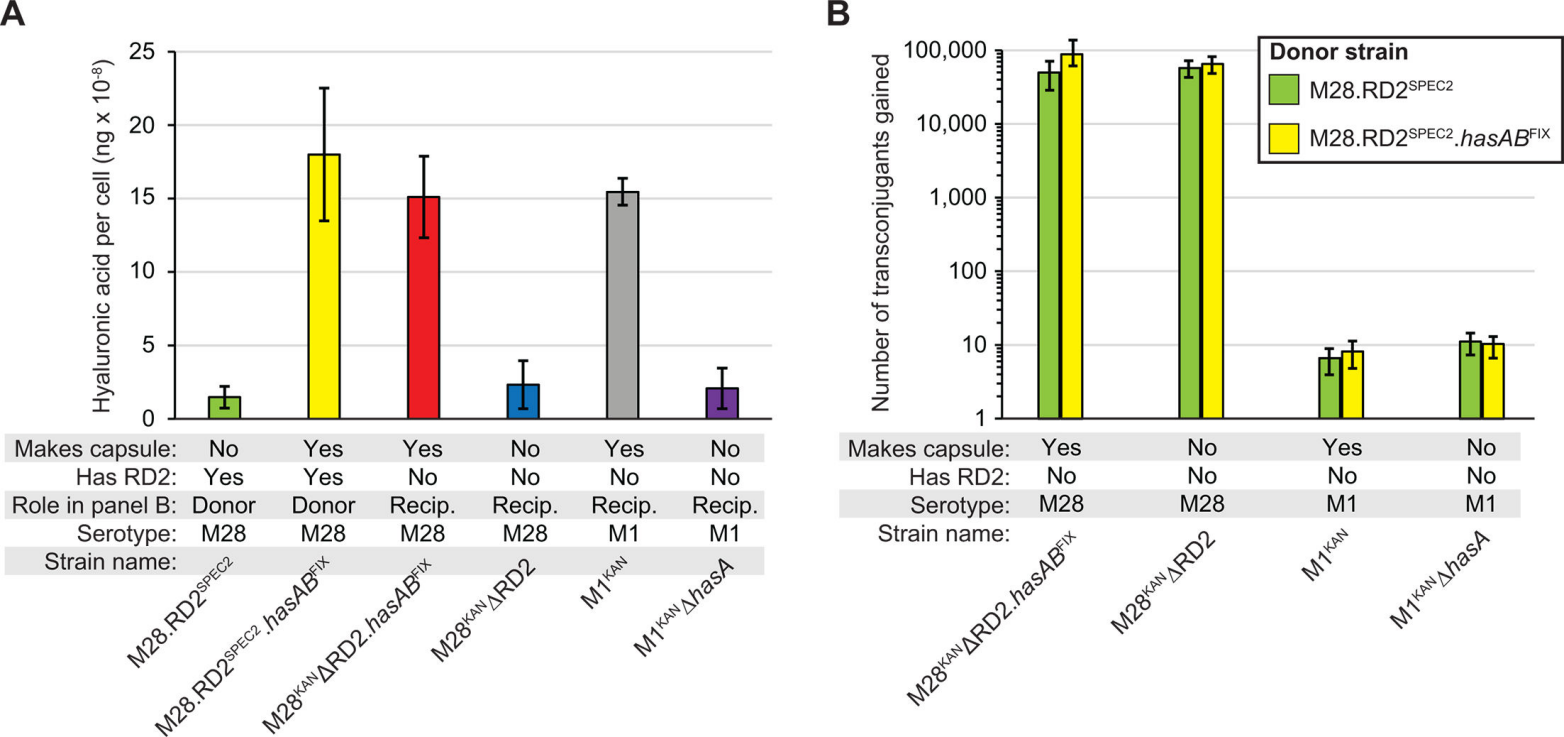
RD2 conjugation is not influenced by the capsule status of the donor or recipient GAS strains. (**A**) Hyaluronic acid capsule assays. Exponential-phase cultures of the indicated GAS strains were analyzed for levels of the hyaluronic acid capsule. The experiment was performed on three occasions, using duplicate cultures of each strain in each experiment, and the values shown are the means ± standard deviations (error bars). Statistical significance was determined by ANOVA, followed by Tukey’s multiple comparisons test. All pairwise comparisons between capsule-producing and noncapsule-producing strains were statistically significantly different (*P* < 0.0001), while there were no statistical differences between any of the capsule-producing strains or between any of the noncapsule-producing strains. (**B**) The M28 GAS strains M28.RD2^SPEC2^ and M28.RD2^SPEC2^.*hasAB*^FIX^ were used as donor strains in intra-serotype (recipient M28 strains M28ΔRD2^KAN^.*hasAB*^FIX^ and M28ΔRD2^KAN^) and inter-serotype (recipient M1 strains M1^KAN^ and M1^KAN^Δ*hasA*) RD2 conjugation assays. The conjugation assays were performed on three separate occasions using two biological replicates on each occasion. Statistical significance was determined by ANOVA, followed by Tukey’s multiple comparisons test. Pairwise comparisons between any of the intra-serotype data versus any of the inter-serotype data identified statistical significance (*P* < 0.0015), while no comparisons were significant testing within the intra-serotype data or within the inter-serotype data.

To test the capsule as a potential barrier for RD2 transfer and acquisition, we generated derivatives of M1 and M28 with varying combinations of the capsule and RD2 presence/absence. All created strains were tested for capsule production via hyaluronic acid capsule assays and matched the expected findings (Fig. 4A). Conjugation assays were set up using two different donors, the acapsular M28.RD2^SPEC2^ and the encapsulated M28.RD2^SPEC2^.*hasAB*^FIX^, and four different recipients, the acapsular M1^KAN^Δ*hasA* and M28^KAN^ΔRD2 and the encapsulated M1^KAN^ and M28^KAN^ΔRD2.*hasAB*^FIX^. In line with the initial study (Fig. 2), significantly higher numbers of transconjugants were obtained in intra-serotype conjugation compared to inter-serotype conjugation, regardless of the capsule status (Fig. 4B). The data are consistent with capsule not serving as a physical barrier to RD2 transfer for either the donor or the recipient.

### The ability of RD2 to enhance GAS adherence to vaginal epithelial cells is influenced by the capsule

**Fig 5:**
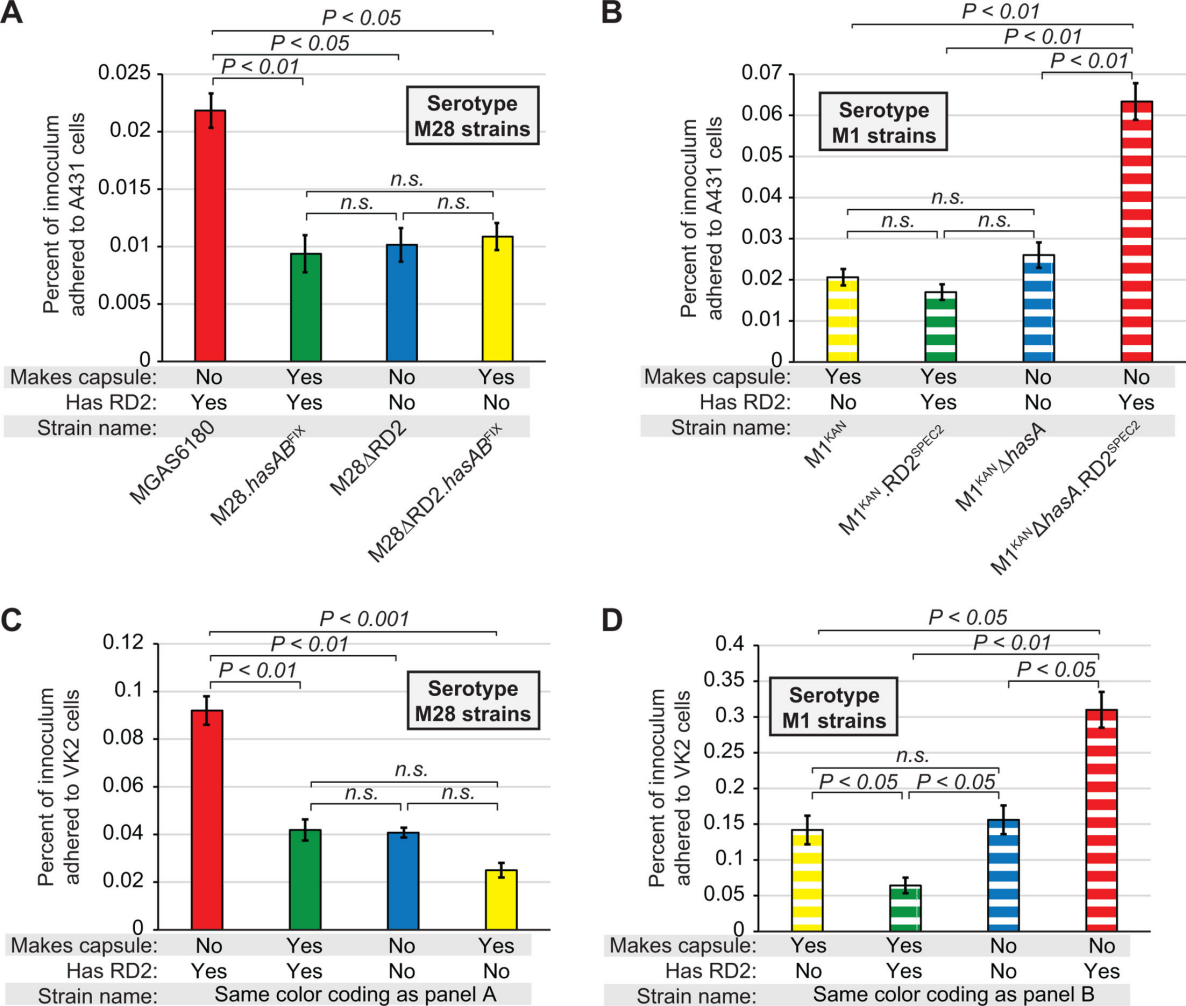
RD2 promotes GAS adherence to human epithelial cell lines in a capsule-dependent manner. (**A–D**) The four indicated M28 (**A and C**) and M1 (**B and D**) strains were compared in tissue culture-based assays of adherence using A431 (**A and B**) and VK2/E6E7 (**C and D**) human cell lines. The assays were performed on a minimum of six occasions with each set of GAS strains, and cell lines and mean values (± standard error of the mean) are shown. Statistical significance was investigated via ANOVA with the Tukey multiple comparisons test, with *P* values shown. NS, not significant.

While our data are consistent with the capsule not blocking RD2 transfer, the capsule may nonetheless indirectly influence the distribution of RD2 in the GAS population by inhibiting the activity of RD2-encoded factors and hence reducing positive selection pressures associated with RD2 gain. As an initial test of this hypothesis, we performed tissue culture-based adherence assays. We utilized two distinct human vaginal epithelial cell lines, VK2/E6E7 and A431, to ensure that our observations were not cell line-specific. The data obtained from each cell line were essentially identical and indicated that RD2 enhanced GAS adherence only in the absence, not in the presence of the capsule (Fig. 5). The data reinforce our prior findings regarding RD2’s ability to promote GAS adherence to human vaginal epithelial cell lines (10, 13), while newly discovering that the adherence-enhancing activity of RD2 only occurs, at least in M1 and M28 strain backgrounds, in strains that lack the capsule.

### The ability of RD2 to enhance GAS colonization in a mouse model of vaginal colonization is not influenced by the capsule

**Fig 6:**
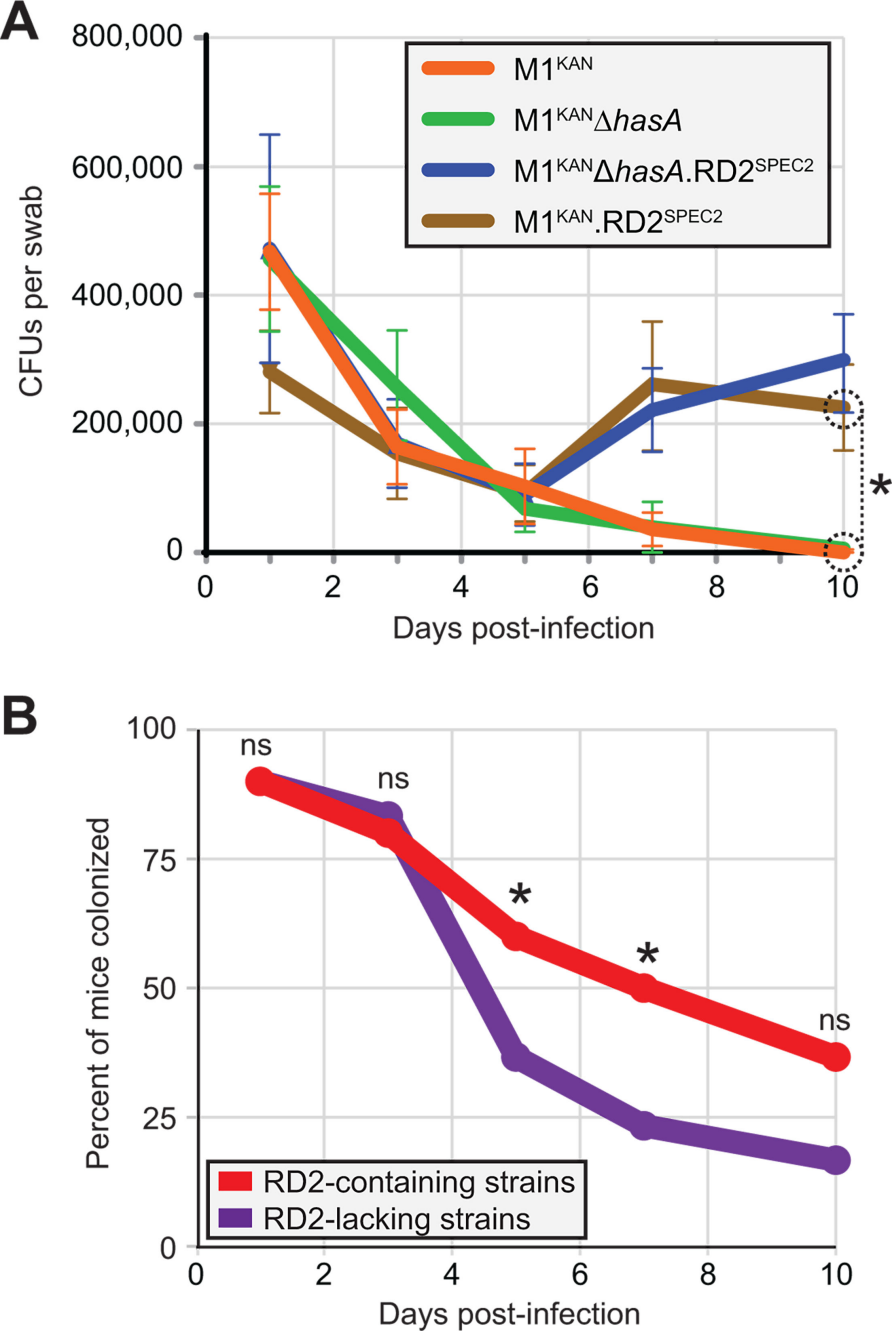
RD2, but not capsule, impacts vaginal colonization by GAS in a mouse model of infection. Groups of 15 estradiol-treated mice (0.5 mg) were vaginally challenged with 1.1 × 10^4^ CFU of the indicated GAS strains. (**A**) Shown is the mean number (± the standard error of the mean) of CFUs recovered from vaginal swabs over time for the four tested isolates. The data were tested for significance using mixed-effects analysis with Tukey’s multiple comparisons test (via GraphPad Prism 10), identifying significance (asterisk) at the 10-day time point for strain M1^KAN^.RD2^SPEC2^ relative to strains M1^KAN^ (*P* = 0.02) and M1^KAN^Δ*hasA* (*P* = 0.03). No other comparisons were statistically significant. (**B**) Cumulative comparison (i.e., the 30 mice infected with a GAS strain lacking RD2 against the 30 mice infected with a GAS strain that contains RD2) showing the percentage of mice that were colonized over time. Differences between the two data sets were tested for significance at individual time points by Fisher’s exact test (*, *P*  <  0.05; n.s., not significant).

Our previous studies identified that RD2 promotes GAS colonization of the female reproductive tract (10, 13). To test whether capsule production influences this activity, we used a mouse model of vaginal colonization. Four GAS strains, differing in their presence or absence of RD2 and of the capsule, were compared by recovering GAS colony-forming units (CFUs) from vaginal swabs of infected mice over time. We identified statistical significance at the 10-day timepoint for strain M1^KAN^.RD2^SPEC2^ relative to the two RD2-lacking strains (Fig. 6A). While no significance was observed for the second RD2-containing strain, M1^KAN^Δ*hasA*.RD2^SPEC2^, relative to the RD2-lacking strains, there was a trend toward significance at the 10-day timepoint (*P* = 0.071). There was no significance between paired strains that differed by capsule presence or absence (e.g., M1^KAN^ -v- M1^KAN^Δ*hasA; P* > 0.95 at all time points). We also gathered data regarding the percent of mice that were colonized by our GAS strains over time and after grouping strains based upon the RD2 status (presence or absence) identified statistical significance, with a higher percentage of mice colonized by RD2-containing strains (Fig. 6B). No significance was observed with the percent of mice colonization data when strains were grouped by the capsule production status rather than by RD2 presence (data not shown). The data are consistent with our previous findings indicating that RD2 promotes GAS vaginal colonization and newly identifies that this occurs in a capsule-independent manner.

### A chromosomally encoded Type I restriction–modification (R-M) system is a barrier to inter-serotype RD2 acquisition

**Fig 7:**
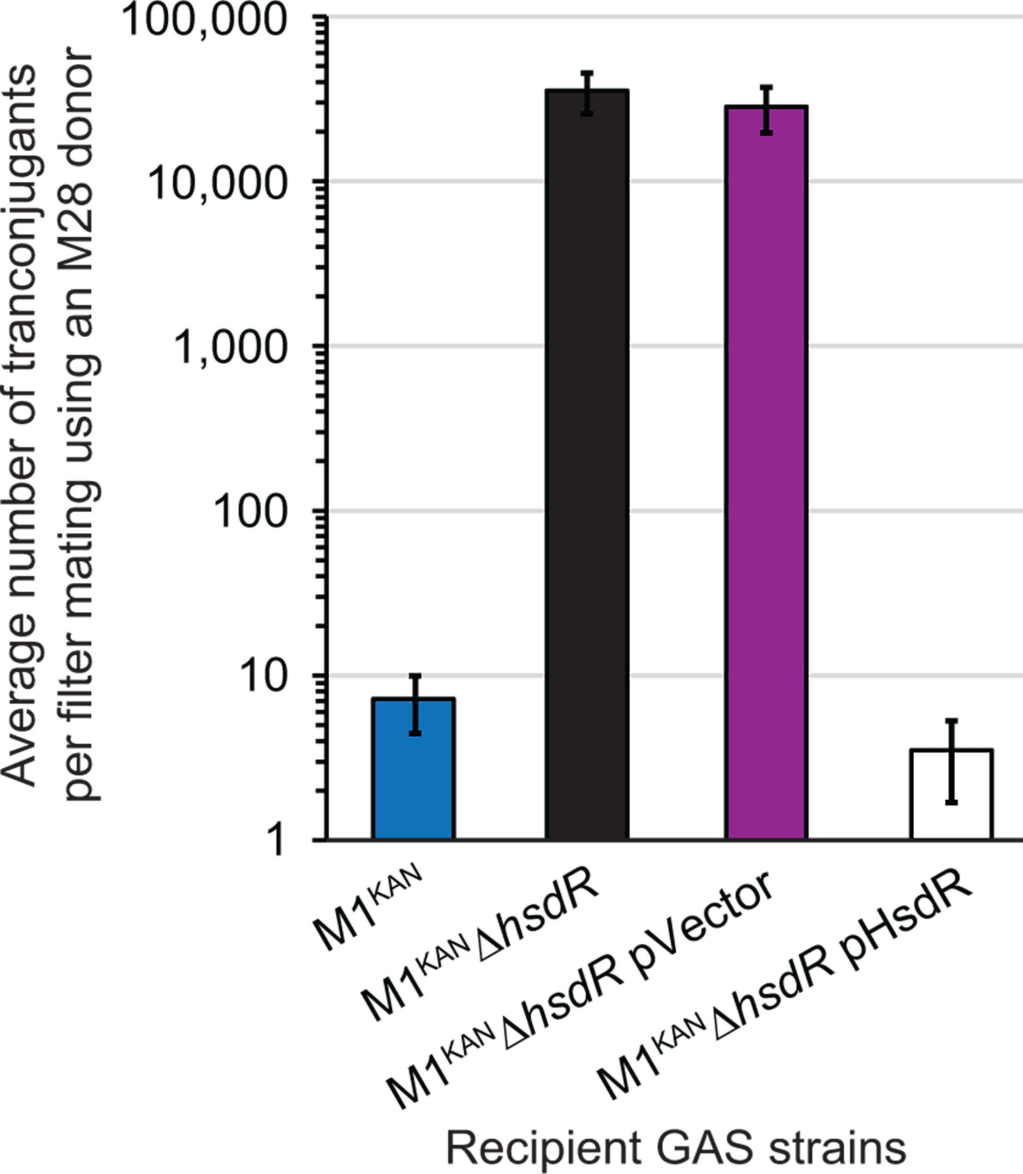
A Type I restriction–modification system represents a major barrier to the inter-serotype conjugation of RD2. RD2 conjugation data gained using the indicated recipient serotype M1 GAS strains and the donor serotype M28 strain M28.RD2^SPEC2^. Data gained with recipient strains M1^KAN^ and M1^KAN^Δ*hsdR* pHsdR, which have an active Type I R-M system, were statistically significantly different relative to recipient strains M1^KAN^Δ*hsdR* and M1^KAN^Δ*hsdR* pVector, which lack a functional Type I R-M system (ANOVA followed by Tukey’s multiple comparisons test; *P* < 0.0001). No other pairwise comparisons were significant.

Most GAS isolates harbor a conserved Type I R-M system that dramatically reduces transformation efficiencies (34). Type I R-M systems consist of three proteins, HsdS which has DNA-binding activity and provides sequence specificity, HsdM which binds with HsdS to make a modification complex capable of methylating DNA at the recognition sequence to enable distinction between self-DNA (methylated) and non-self-DNA (not methylated), and HsdR which complexes with HsdSM to form a restriction complex that cleaves DNA up to 1 kb away from unmodified recognition sequences (35). The recognition sequence recognized by the GAS Type I R-M system differs between, and in rare cases within, serotypes (36, 37). Thus, while not previously tested with regards to impacting conjugation frequencies, this R-M system may be behind the low number of transconjugants from our inter-serotype conjugation assays (Fig. 2C). To test the contribution of the Type I R-M system as a barrier to the inter-serotype gain of RD2, we created an M1 derivative strain that lacks *hsdR* (strain M1^KAN^Δ*hsdR*) and hence does not produce the restriction subunit required for cleavage. This strain, along with a plasmid-complemented derivative, and others, were used in conjugation assays. The two tested *hsdR* mutant derivative strains yielded orders of magnitude higher numbers of transconjugants compared to the parental and complemented mutant strains (Fig. 7), reaching the levels seen in our intra-serotype assays (Fig. 2C). The data are consistent with those of the conserved Type I R-M system representing a major barrier to the intra-serotype spread of RD2.

### RD2 modifies the GAS transcriptome in an *ex vivo* model of invasive infection

**Fig 8:**
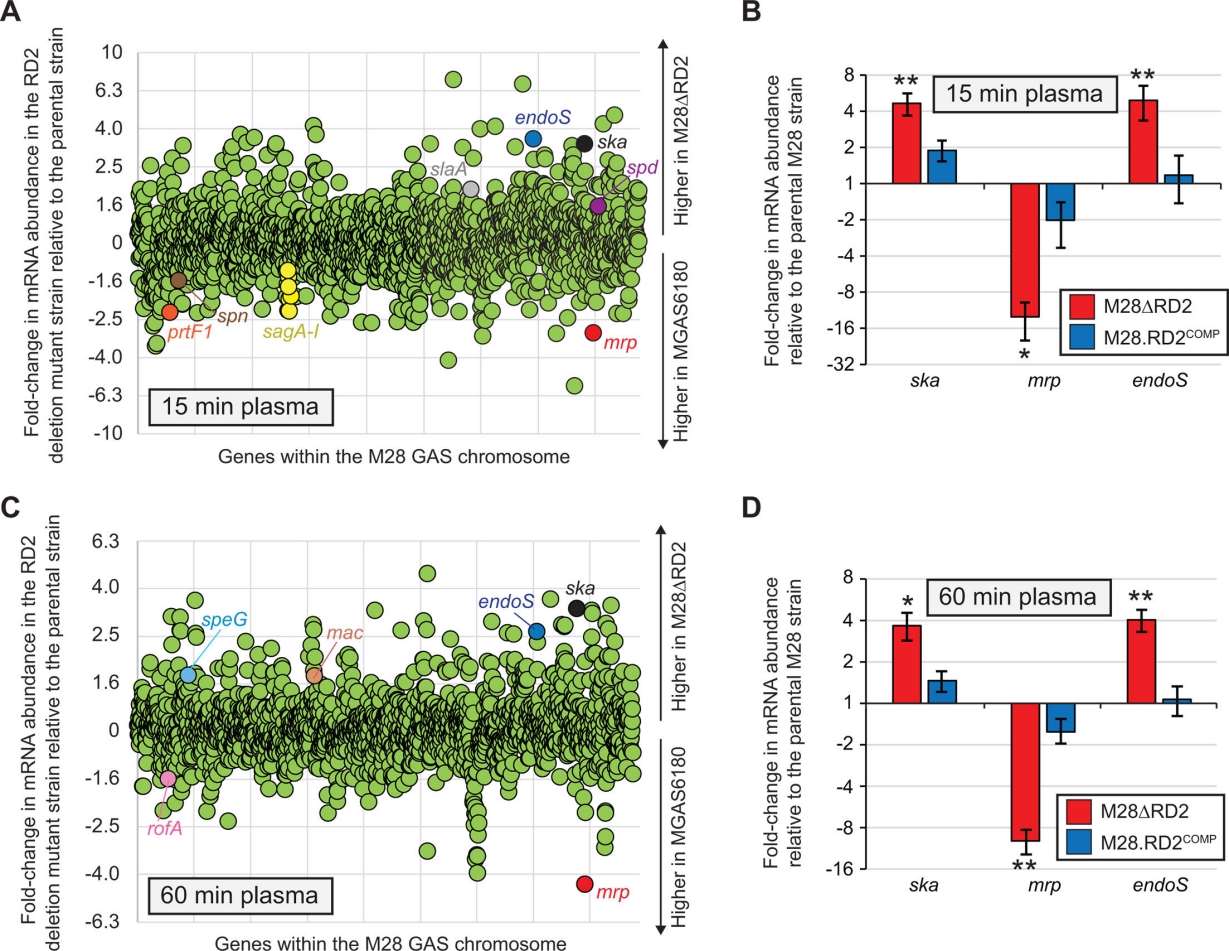
Influence of RD2 on the GAS transcriptome following exposure to human plasma. RNA-Seq analysis comparing the parental M28 strain MGAS6180 and its RD2-deletion mutant derivative (M28ΔRD2) following 15 minutes (**A**) or 60 minutes of (**C**) exposure to human plasma. Each circle represents mRNA from a single gene. Genes are arranged in the same order as they appear in the chromosome. Select virulence factor-encoding mRNAs are colored and labeled. (B/D) TaqMan-based quantitative RT-PCR data verifying the differential regulation of select virulence factor-encoding genes between the presence and absence of RD2. The abundance of the indicated mRNAs was determined from triplicate cultures of the two strains following 15 minutes (**B**) or 60 minutes of (**D**) exposure to human plasma. The experiment was performed in triplicate with mean (± standard deviation) shown. Statistical significance was investigated via two-way ANOVA with the Tukey multiple comparisons test (asterisks highlight significance relative to the parental M28 isolate; *, *P* < 0.01; **, *P* < 0.001).

GAS can be exposed to plasma during infection, for example, by vascular leakage or following entry into the bloodstream (38, 39). We used GAS exposure to human plasma, an *ex vivo* model of an invasive infection, to test the ability of RD2 to modify the GAS transcriptome, building upon our previous data using *in vitro* grown samples (10). In part, this experiment was performed to assess potential transcriptional changes that may negatively impact the fitness of RD2-containing GAS strains, which could be a factor in the maintenance of RD2 within GAS populations. The parental M28 isolate MGAS6180 and its RD2 deletion mutant derivative M28ΔRD2 were grown to the exponential phase of growth in THY broth before washing the cells and resuspending in human plasma. Samples were recovered for RNA-Seq analysis 15 minutes and 60 minutes following plasma exposure. A total of 404 genes for the 15-minute samples and 219 genes for the 60-minute samples were identified as being statistically significantly expressed at a minimum of 1.5-fold cutoff level (Fig. 8A and C; Tables S2 and S3). Included within the differentially regulated genes were multiple virulence factor-encoding genes, including those encoding the thrombolytic agent streptokinase (*ska*) (40); the M-related protein (*mrp*), which is a fibrinogen-binding adhesin with anti-phagocytic activity (41); and the IgG endoglycosidase (*endoS*) (42). Verification of the differential transcript levels for these three genes was gained by quantitative real-time (RT)-PCR comparing the parental, RD2 deletion mutant, and complemented mutant strains (Fig. 8B and D). The data are consistent with the observation of RD2 directly or indirectly influencing the GAS transcriptome during infection.

## DISCUSSION

Serotype M28 isolates cause most GAS puerperal sepsis cases (43). A contributing factor in this GAS–serotype disease–phenotype association is the presence of the 36-kb RD2 pathogenicity island within the genomes of M28 isolates, but not within the genomes of most other GAS serotypes (10, 44). We previously identified that RD2 enhances the ability of GAS to colonize the female reproductive tract and, in serotype-specific manners, promote adherence to vaginal epithelial cells and survival in human blood (10, 13). Given the activities bestowed by RD2, it is perhaps therefore surprising that its presence is restricted to M28 isolates and a subsection of M2 and M77 isolates. In this study, we investigated reasons for the limited prevalence of RD2 in the GAS population, with possibilities ranging from serotype-specific barriers to RD2 acquisition to serotype-specific differences in selection pressures following RD2 transfer. A major focus of the work involved investigation of the role of the capsule in the serotype-specific distribution of RD2. Capsules play critical immune evasion roles in numerous bacterial and fungal pathogens (30, 45). In addition, their extracellular location surrounding and masking the microbial surface results in capsules, at least in some instances, reducing the conjugation frequency (27). We identified that the presence of a capsule did not appreciably impact the donation or receipt of RD2 (Fig. 4), nor did it impact the ability of RD2 to promote vaginal colonization (Fig. 6). Capsule did, however, interfere with the ability of RD2 to promote GAS adherence to two vaginal epithelial cell lines (Fig. 5). However, the consequence of capsule production on RD2 function appears to be serotype-specific, as a previous study in which we introduced RD2 into encapsulated M49 and M59 strains identified that RD2 enhanced both vaginal colonization and vaginal epithelial cell adherence (13). Thus, serotype-specific differences (e.g., in the amount of the capsule made or in the production of other factor/s) impact the functional consequences of RD2 gain. If the positive consequences of harboring RD2 are inhibited by the capsule, they may not outweigh the energy burden of maintaining this element, which could lead to RD2 loss (46, 47).

We are the first to generate and study capsule-producing serotype M28 GAS strains, with capsule production being visualized via India ink stain (Fig. 3C) and quantified by hyaluronic acid enzyme-linked immunosorbent assay (ELISA) (Fig. 3B). Note that we did not directly assess the form of hyaluronic acid produced by our M28 strains, but are working under the assumption is that it is the high-molecular-weight form that has been identified in other GAS serotypes (48–50). In the process of generating our capsule-producing serotype M28 GAS strains, we identified that the acapsular phenotype of natural M28 isolates is not a consequence of a single mutation within the *has* operon. A 1-bp insertion within *hasA* likely results in an inactive protein; however, removal of the insertion, while also converting the E178K and I373V *hasA* changes and the Y52D *hasB* change to those observed in M1 alleles (Fig. 3A), failed to restore capsule production (data not shown). In contrast, additionally altering the I10V and K60N changes in *hasA*, and the A207D and Y241H changes in *hasB*, restored capsule production (Fig. 3). Thus, one or more of these amino acid differences in the M28 background must, along with the 1-bp insertion, prevent capsule production.

Part of our motivation to investigate the capsule as a potential barrier to RD2 transfer came from the fact that MGAS10270, the first serotype M2 GAS isolate to be genome-sequenced (51), harbors a likely inactivating mutation (an 11-bp deletion) within *hasB*, while also harboring RD2 (Fig. S1A). As only a subpopulation of M2 isolates harbor RD2, this led us to hypothesize that M2 isolates consisted of a mixture of encapsulated isolates that lack RD2 and non-encapsulated isolates that contain RD2. We investigated this by examining nine additional M2 GAS isolates for the presence/absence of the 11-bp deletion in *hasB* and of RD2. PCR analysis identified that all nine isolates harbored RD2 (Fig. S1B and C), while a targeted sequencing approach identified that none of the isolates had any obvious inactivating mutations in the *has* operon (data not shown). Therefore, the presence/absence of RD2 in M2 GAS isolates is unlikely to track with capsule production.

Why we observed differences between our colonization and adherence data regarding the impact of the capsule on the RD2-mediated enhancement of these phenotypes is unknown. Possibilities include differential gene expression, both within and outside of RD2, under the conditions used in these assays, and phenotype-specific activities of RD2 encoded proteins. Two known adhesion-encoding genes are located within RD2, *aspA* and *r28* (52–54), while others are hypothesized (Fig. 1). The large size (~1,400 amino acids) of R28 and AspA may enable their extension above the capsule layer and maintain activity in the presence of the capsule, potentially providing an explanation for why the capsule did not impact vaginal colonization in our mouse model. In contrast, we previously identified that R28 has no role in adherence to the tested cell lines (10), while we have not tested AspA. Thus, if these proteins play no role in adherence to A431 or VK2 cells, it may be one of the smaller, predicted RD2 adhesins (i.e., Spy1306 or Spy1326) that play a role, and these may be inhibited by the capsule. On a related note, it should be mentioned that no RD2-encoded gene was identified from a transposon mutagenesis screen as promoting vaginal colonization in a nonhuman primate model of infection (55). However, given that RD2 exists in ~eight copies per cell, it is unlikely that the mutagenesis process used would have mutated more than a single copy of any given RD2-encoded gene, and hence the value of the study’s findings with regards to RD2 is unclear (19).

We gained a ~ 10,000 fold higher number of transconjugants following intra-serotype conjugation assays than following inter-serotype assays (Fig. 2). After finding that capsule production was not responsible (Fig. 4), we turned our attention to the conserved Type I R-M system (34, 36, 37). The specificity of this system is controlled by the HsdS subunit via two distinct target recognition domains (TRDs), each of which binds to half of the recognition sequence. Thirteen distinct GAS TRD combinations have been identified to date, with M28 isolates such as MGAS6180 harboring the TRD_DA_ combination in HsdS, which detects the sequence 5’- CRAAnnnnnnTGC −3’ (and its corresponding partner motif 5’- GCAnnnnnnTTYG −3’) (37, 56). In contrast, HsdS from our M1 isolate MGAS2221 harbors TRD_AG,_ which detects the sequence GCAnnnnnnTTAA (and corresponding partner motif TTAAnnnnnnTGC) (37). The recognition motifs of the additional strains used in our conjugation assays (Fig. 2C; PGAS696 [M6], 591 [M49], and MGAS15252 [M59]) have not been experimentally determined, but based upon the sequence of HsdS harbor TRD_DF_, TRD_CF_, and TRD_DA_, respectively. Of note, for all the known or predicted recognition motifs for our tested strains, there are multiple (e.g., three for TRD_DA_ [M28] and nine for TRD_AG_ [M1]) within RD2. The differing recognition sequences between the Type I R-M system in our donor M28 strain and recipient M1, M6, and M49 strains likely explain the importance of this system in inhibiting the conjugative transfer of RD2. However, our M28 and M59 GAS isolates both share TRD_DA_, meaning that they should share the same recognition sequence, and hence there should not be the barrier to RD2 conjugation that we observed. While sharing the same TRDs, the HsdS proteins of M28 and M59 isolates are not identical, differing in four amino acids. As the recognition motif of the M59 Type I R-M system has not been experimentally determined, it is possible that the sequence variation between the HsdS proteins leads to an altered motif. Additional explanations include a potential additional, and uncharacterized, R-M system within our M59 strain, or some strain- or serotype-specific activity related to the resident CRISPR-Cas systems (57).

Our RNA-Seq data are consistent with the observation of RD2 directly and/or indirectly altering the GAS transcriptome during infection (Fig. 8). While not assessed in this study, direct transcriptome modulation by RD2 may occur through the action of the one known and four putative transcription factors encoded by this island (Fig. 1) (58). Indirect transcriptome modulation may occur due to the well-described intersection between metabolism and transcription (59, 60), with the energy burden placed on the cell due to the presence of RD2 possibly playing a role. Our transcriptome data represent the first *ex vivo* analysis of the consequences of RD2 presence and relative to our previous *in vitro* data show similarities, e.g., increased transcripts of the adhesin-encoding gene *mrp*, and differences, e.g., lower transcripts of *ska* in the presence of RD2 (13, 61). The finding that RD2 enhances adhesin gene transcript levels is consistent with our animal data showing that RD2 enhances vaginal colonization (Fig. 6B) (10, 13).

In summary, we have identified that the conserved Type I R-M system encoded by most GAS isolates represents a major barrier to the acquisition of RD2, providing an explanation for the observation of RD2 being restricted to a select number of GAS serotypes. This represents the first experimental evidence that the Type I R-M system reduces the frequency of conjugation in GAS, as it does to the transformation frequency (34). Given the disease-modulating activities of RD2, and its association with cases of puerperal sepsis, further research into the activity and distribution of this mobile genetic element is warranted.

## MATERIALS AND METHODS

### Bacterial strains and growth conditions

The GAS strains used in this study are listed in Table 1. GAS strains were cultured in Todd–Hewitt broth that contains 2% yeast extract (THY broth) with kanamycin (300 µg/mL), erythromycin (1 µg/mL), and spectinomycin (150 µg/mL) added as needed.

**TABLE 1:** GAS strains used in this study^*a*^

GAS strain	Description	Reference
MGAS6180	A well-characterized clinical serotype M28 isolate	(9)
MGAS2221	A well-characterized clinical serotype M1 isolate	(6)
PGAS696	A clinical serotype M6 isolate	This work
591 (aka. PGAS812)	A well-characterized clinical serotype M49 isolate	(62)
MGAS15252	A well-characterized clinical serotype M59 isolate	(31)
M6^KAN^ (aka. PGAS1146)	PGAS696 derivative with a kanamycin resistance cassette inserted in the intergenic region downstream of the *treR* gene	This work
M49^KAN^ (aka. PGAS1147)	PGAS812 derivative with a kanamycin resistance cassette inserted in the intergenic region downstream of the *treR* gene	This work
M59^KAN^ (aka. PGAS1145)	MGAS15252 derivative with a kanamycin resistance cassette inserted in the intergenic region downstream of the *treR* gene	This work
M1Δ*hsdR* (aka. PGAS881)	MGAS 2221 derivative in which the *hsdR* gene has been replaced with an erythromycin resistance cassette	This work
M1^KAN^Δ*hsdR* (aka. PGAS 1148)	M1Δ*hsdR* derivative with a kanamycin resistance cassette inserted in the intergenic region downstream of the *treR* gene	This work
M1Δ*hsdR* pVector (aka. PGAS944)	M1Δ*hsdR* derivative containing the empty vector pDCBB	This work
M1^KAN^Δ*hsdR* pVector (aka. PGAS 1149)	PGAS944 derivative with a kanamycin resistance cassette inserted in the intergenic region downstream of the *treR* gene	This work
M1Δ*hsdR* pHsdR (aka. PGAS970)	M1Δ*hsdR* derivative containing the wild-type *hsdR* gene from M1 GAS on a pDC123-based plasmid	This work
M1^KAN^ΔhsdR + pHsdR (aka. PGAS 1150)	PGAS970 derivative with a kanamycin resistance cassette inserted in the intergenic region downstream of the *treR* gene	This work
M28.RD2^SPEC2^ (aka. PGAS1000)	MGAS6180 derivative in which a spectinomycin resistance cassette has been inserted into RD2	This work
M1^KAN^ (aka. PGAS40)	MGAS2221 derivative with a kanamycin resistance cassette inserted in the intergenic region downstream of the *treR* gene	(63)
M28ΔRD2 (aka. PGAS862)	MGAS6180 derivative in which RD2 has been deleted	(10)
M1^KAN^Δ*hasA* (aka. PGAS1025)	M1^KAN^ derivative in which the *hasA* gene has been replaced with an erythromycin resistance cassette	This work
M28.*hasAB*^FIX^ (aka. PGAS1010)	MGAS6180 derivative that harbors *hasAB* alleles identical to M1 isolates	This work
M28.RD2^SPEC2^.*hasAB*^FIX^ (aka. PGAS1117)	M28.*hasAB*^FIX^ derivative in which a spectinomycin resistance cassette has been inserted into RD2	This work
M28ΔRD2.*hasAB*^FIX^ (aka. PGAS1038)	M28ΔRD2 derivative that harbors *hasAB* alleles identical to M1 isolates	This work
M28^KAN^ΔRD2.*hasAB*^FIX^ (aka. PGAS1040)	M28ΔRD2.*hasAB*^FIX^ derivative with a kanamycin resistance cassette inserted in the intergenic region downstream of the *treR* gene	This work
M28^KAN^ΔRD2 (aka. PGAS1024)	M28ΔRD2 derivative with a kanamycin resistance cassette inserted in the intergenic region downstream of the *treR* gene	This work
M1^KAN^.RD2^SPEC2^ (aka. PGAS1031)	M1^KAN^ derivative in which RD2^SPEC2^ has been conjugated into it from M28.RD2^SPEC2^	This work
M1^KAN^Δ*hasA*.RD2^SPEC2^ (aka. PGAS1029)	M1^KAN^Δ*hasA* derivative in which RD2^SPEC2^ has been conjugated into it from M28.RD2^SPEC2^	This work
M28.RD2^COMP^ (aka. PGAS863)	Complemented derivative of M28ΔRD2 in which RD2 has been re-introduced	(10)
MGAS10270	Clinical M2 GAS isolate that has been whole-genome-sequenced and was isolated in the late 1990 s from a patient with pharyngitis in TX	(51)
PGAS904 (aka. ABC020017475)	Clinical M2 GAS that was isolated in 2003 from the blood of a patient in MN	ABCs/EIP
PGAS905 (aka. ABC020024781)	Clinical M2 GAS that was isolated in 2004 from the blood of a patient in NY	ABCs/EIP
PGAS908 (aka. ABC020041230)	Clinical M2 GAS that was isolated in 2007 from the wound of a patient in MD	ABCs/EIP
PGAS909 (aka. ABC020041415)	Clinical M2 GAS that was isolated in 2008 from the blood of a patient in MN	ABCs/EIP
PGAS910 (aka. ABC020048910)	Clinical M2 GAS that was isolated in 2009 from the blood of a patient in NY	ABCs/EIP
PGAS911 (aka. ABC020052356)	Clinical M2 GAS that was isolated in 2010 from the blood of a patient in GA	ABCs/EIP
PGAS912 (aka. ABC020057330)	Clinical M2 GAS that was isolated in 2011 from the blood of a patient in GA	ABCs/EIP
PGAS913 (aka. ABC020060776)	Clinical M2 GAS that was isolated in 2012 from the blood of a patient in TN	ABCs/EIP
PGAS914 (aka. ABC020073981)	Clinical M2 GAS that was isolated in 2014 from the blood of a patient in NM	ABCs/EIP

^*a*^ ABCs/EIP refers to the fact that these isolates were provided for this study by the Active Bacterial Core surveillance (ABCs)/Emerging Infections Programs (EIP) Network.

### Creation of GAS strain M28.RD2^SPEC2^

We created this strain to serve as a donor in conjugation assays. This strain was created by tagging RD2 with an antibiotic resistance cassette. Flanking sequences (~ 0.8 kb in size) upstream and downstream of the partial transposase-encoding gene (M28_Spy1335; aka M28_RS06845) within RD2 were joined by overlap extension PCR on either side of a spectinomycin resistance cassette (~ 1 kb) using primers mentioned in Table S1. The resulting sequence was cloned into the pCR2.1 TOPO vector (Thermo Fisher). After sequence verification, the constructed plasmid was digested with *Eco*RV, the ~2.5-kb insert gel purified, and used to transform competent MGAS6180 cells. Following electroporation, transformants were selected on THY agar plates containing spectinomycin. The constructed strain, M28.RD2^SPEC2^, was verified by PCR and targeted sequencing.

### Filter mating (conjugation) assay

GAS strains (donors and recipients) were streaked onto THY agar plates that contained the required strain-specific antibiotics. Overnight cultures of the donors and recipients were set in 10 mL THY broth with the appropriate antibiotics and then used to inoculate 35 mL of fresh day cultures (three cultures per strain and different amounts of the overnight were added to each). Strains were grown until one of the cultures for each of the tested strains reached an O.D_600_ of 0.2. These cultures were then mixed in an equal volume (30 mL of the donor and 30 mL of the recipient) and collected on the surface of a Nalgene sterile nitrocellulose filter (pore size of 0.45 µm). The filter was subsequently transferred to a blood agar plate and incubated at 37°C for between 0.5 and 8 hours (filter mating time) in a 5% CO_2_ incubator. After incubation, the filter was removed, and the side harboring the bacteria (which had been in contact with the agar plate) was scraped and washed with 1 mL sterile PBS. The bacteria collected in this 1 mL of PBS were serially diluted, and 100 µL of undiluted and 1:10, 1:100, and 1:1000 dilutions were plated on THY agar plates containing kanamycin and spectinomycin. The plates were then incubated at 37°C for 16 hours in a 5% CO_2_ incubator. The number of transconjugants was calculated for each set of donor and recipient pairs. For each donor–recipient pair, three random colonies resistant to the selected antibiotics were chosen and checked for their recipient background by PCR, like that shown in Fig. 2B.

### Creation of a library of additional GAS strains to serve as recipients in conjugation assays

Kanamycin-resistant derivatives of the GAS isolates PGAS696 (creating strain M6^KAN^), M28ΔRD2 (creating strain M28^KAN^ΔRD2), 591 (creating strain M49^KAN^), and MGAS15252 (creating strain M59^KAN^) were created for use, alongside the previously created strain M1^KAN^, as recipient strains in conjunction assays. The kanamycin resistance gene from strain M1^KAN^ was PCR-amplified and transformed into competent cells of the four GAS isolates (see Table S1 for primer sequences). Following electroporation, transformants were selected on THY agar plates containing kanamycin. The constructed strains were verified by PCR and targeted sequencing.

### Creation of GAS strain M1^KAN^Δ*hasA*

We created this strain to serve as a capsule-negative control in the M1 background. This strain was created by replacing the capsule biosynthesis gene *hasA* with an antibiotic resistance cassette. The upstream and downstream regions (~0.9 kb) of *hasA* were PCR-amplified using the primers listed in Table S1. The purified PCR products were joined by overlap PCR to each side of a PCR-amplified erythromycin-resistant cassette. The resulting PCR product was cloned into the pCR2.1 TOPO vector (Thermo Fisher) and sequence-verified. The insert was digested out of the plasmid, gel-purified, and transformed into GAS strain M1^KAN^. Following electroporation, transformants were selected on THY agar plates containing kanamycin and erythromycin. The constructed strain, M1^KAN^Δ*hasA*, was verified by PCR and targeted sequencing. The absence of transcriptional polar effects on the downstream *hasBC* genes was confirmed by qRT-PCR analysis (data not shown).

### Creation of GAS strain M28.*hasAB*^FIX^ and its derivative M28.RD2^SPEC2^.*hasAB*^FIX^

We created strain M28.*hasAB*^FIX^ so that we could assess the consequences of restoring capsule production in the M28 GAS background. To make strain M28.*hasAB*^FIX^, we used an allelic exchange-based method using the suicide vector pBBL740, similar to that which we have previously described (12, 40). The functional *hasAB* genes from the M1 strain MGAS2221 were PCR-amplified and cloned, via Gibson assembly, into PCR-amplified pBBL740 (see Table S1 for the primers used). The plasmid insert was sequence-verified and transformed into MGAS6180-competent cells. Following electroporation, transformants were selected on THY agar plates containing chloramphenicol. Passaging of transformants was performed in THY broth without antibiotics, and chloramphenicol-sensitive colonies were identified through patch plating onto both THY agar and THY agar containing chloramphenicol. Sensitive strains were verified through targeted PCR and sequencing to identify a strain that harbored the corrected *hasAB* genes.

We created strain M28.RD2^SPEC2^.*hasAB*^FIX^ so that this capsule producing M28 strain could be used as a donor strain in conjugation assays. M28.RD2^SPEC2^.*hasAB*^FIX^ is a derivative of M28.*hasAB*^FIX,^ in which we tagged RD2 by inserting a spectinomycin resistance cassette, as described above for the making of strain M28.RD2^SPEC2^. The constructed strain was verified by PCR and targeted sequencing.

### Hyaluronic acid capsule assay

Assessment of GAS strain hyaluronic acid production was performed using the Corgenix hyaluronic acid (HA) test kit, as described previously (64). Briefly, overnight GAS cultures were set in 10 mL THY broth with the appropriate antibiotics. Fresh 35 mL day cultures were set using 350 µL of overnight culture and grown until the OD_600_ reached ~0.6, at which time, 1 mL of the culture was taken per strain, pelleted, washed with 1 mL PBS, and re-suspended in 500 µL water. A 100-µL aliquot of the re-suspended culture was serially diluted, and dilutions 1:100,000 and 1:1,000,000 were plated on blood agar plates to enumerate the inoculum. To remove the capsule, the remaining re-suspended culture was added into a 2-mL screw cap tube containing 1 mL of chloroform. Cells were processed in a FastPrep machine (MP Biomedicals) at a speed of 4.5 for 1 minute and were kept on ice for 1 minute, followed by reprocessing in the FastPrep machine. The lysate was then centrifuged at 13,000 g for 10 minutes, and the aqueous solution was transferred to a clean tube. The hyaluronic acid content was determined using the Corgenix ELISA-based HA test kit in accordance with the manufacturer’s instructions.

### Capsule staining

We followed the method of Loh and colleagues (65). Briefly, exponential-phase bacteria were mixed with Indian ink (Hardy Diagnostics), smeared onto a microscope slide, and allowed to air-dry. The slide was then stained with crystal violet for 1 minute before rinsing with water. Slides were examined and imaged at 1,000 x magnification on a Nikon Eclipse microscope for the presence of capsule, as indicated by clear zones surrounding the cells. Representative images were gained from a minimum of three slides prepared on different days for each strain tested.

### Creation of GAS strain M28ΔRD2.*hasAB*^FIX^ and its derivative M28^KAN^ΔRD2.*hasAB*^FIX^

We created strain M28ΔRD2.*hasAB*^FIX^ as a step toward making an M28 capsule-producing recipient strain for use in conjugation assays. The naturally mutant *hasAB* genes of the previously created, RD2-lacking, M28 strain M28ΔRD2 (10) were replaced with the functioning *hasAB* genes from MGAS2221 as described above for the creation of strain M28.*hasAB*^FIX^. The constructed strain was verified by PCR and targeted sequencing.

We created strain M28^KAN^ΔRD2.*hasAB*^FIX^ for use as an M28 capsule-producing recipient strain in conjugation assays, and this strain is a derivative of strain M28ΔRD2.*hasAB*^FIX,^ which has been tagged by the insertion of a kanamycin resistance gene into the genome. The kanamycin resistance gene from strain M1^KAN^ was PCR-amplified and transformed into competent cells of strain M28ΔRD2.*hasAB*^FIX^ (see Table S1 for primer sequences). Following electroporation, transformants were selected on THY agar plates containing kanamycin. The constructed strain was verified by PCR and targeted sequencing.

### Creation of the GAS strains M1^KAN^.RD2^SPEC2^ and M1^KAN^Δ*hasA*.RD2^SPEC2^

We created strain M1^KAN^.RD2^SPEC2^ following the conjugation of RD2 into strain M1^KAN^. RD2 was conjugated from donor strain M28.RD2^SPEC2^ into strain M1^KAN,^ as described in the filter mating (conjugation) protocol outlined above. The constructed strain was verified by PCR and targeted sequencing. We created strain M1^KAN^Δ*hasA*.RD2^SPEC2^ following the conjugation of RD2 into strain M1^KAN^Δ*hasA*. RD2 was conjugated from donor strain M28.RD2^SPEC2^ into strain M1^KAN^Δ*hasA,* as described in the filter mating (conjugation) protocol outlined above. The constructed strain was verified by PCR and targeted sequencing.

### Tissue culture adherence assay

The assay was performed as mentioned in our previous studies (13). Briefly, the human epithelial cell line VK2/E6E7 was grown in keratinocyte serum-free medium (KSF; Gibco) containing 0.1 ng/mL of human recombinant epidermal growth factor 1–53 (EGF 1–53), calcium chloride (final concentration, 0.4 mM), 0.5 mg/mL bovine pituitary extract, streptomycin (50 µg/mL), and penicillin (50 U/mL). The A431 human epithelial cell line was grown in Dulbecco’s modified Eagle medium (DMEM) containing 10% fetal bovine serum along with streptomycin (50 µg/mL) and penicillin (50 U/mL). At 90% confluency, VK2/E6E7 and A431 cells were resuspended in their respective media but without antibiotics and seeded into wells of a 12-well tissue culture plate. The seeded plates were then incubated at 37°C for 24 hours in a 5% CO_2_ incubator. GAS strains used for the assay were grown to an OD_600_ of 0.4; 1 mL of the culture was then pelleted. The pelleted cells were resuspended in 1 mL of PBS and 100 µL were used to inoculate one or more wells of the seeded 12-well culture plates (MOI ~ 50). Plates were incubated for 5 minutes at 37°C in a 5% CO_2_ incubator. At the same time, 100-µL samples of each PBS-diluted GAS culture (1:1,000,000) were plated onto blood agar plates to enumerate the initial GAS inoculum accurately. After 5 minutes, the liquid in the wells was removed, and the wells were washed four times with 1 mL PBS. After washing, 1% freshly prepared saponin was added to each well and incubated at room temperature for 15 minutes to lyse the tissue culture cells. The lysed tissue culture cells and adhered GAS cells were recovered from the wells by scraping. The lysates obtained were serially diluted up to 1:1000, and 100 µL (from 1:100 and 1:1000) was plated onto the blood agar plates in duplicate. The average bacterial count and the percentage of GAS adhered to the epithelial cells relative to the initial inoculum size were calculated. Statistical significance was determined via ANOVA with the Tukey multiple comparisons test.

### Mouse model of vaginal colonization

A mouse model of vaginal colonization was used as we have previously described (13). In brief, 2 days before streptococcal infection, female CD-1 mice were estrogenized via an intraperitoneal injection of 0.5 mg β-estradiol 17-valerate (Sigma-Aldrich) dissolved in 0.1 mL of sterile sesame oil (Sigma-Aldrich). On the day of inoculation (day 0), the mice were vaginally challenged with 10 µL of a GAS suspension with 1.1 × 10^4^ CFU as the inoculum. Each tested GAS strain was used to infect fifteen mice. Colonization levels were determined on days 1, 3, 5, 7, and 10 through vaginal swabs. These swabs were vortexed in 1 mL of PBS, the PBS was serially diluted, and 100 µL of the neat (undiluted), 10^−2^ dilution, and 10^−4^ dilution were plated onto Selective Strep agar plates with 5% sheep blood (Thermo Scientific Strep Selective II Agar). Plates were incubated overnight at 37°C with 5% CO_2_ and the number of CFUs calculated. Our limit of detection in these studies was 10 CFUs per vaginal swab. This experiment complied with the guidelines specified in a protocol approved by the Institutional Animal Care and Use Committee of the University of Nevada, Reno.

### Creation of a *hsdR* mutant derivative of GAS strain MGAS2221

To facilitate testing the contribution of the chromosomally encoded Type I R-M system to the observed barrier to RD2 conjugation seen in our tested M1 isolate (Fig. 2A), we created a *hsdR* mutant derivative of strain MGAS2221. 1-kb regions flanking *hsdR* were PCR-amplified (see Table S1 for primer sequences), inserted on either side of a PCR-amplified erythromycin resistance gene via overlap extension PCR, and the resultant 3-kb product was cloned into the pCR2.1 TOPO vector (Thermo Fisher). After sequence verification, we digested the plasmid, gel extracted the insert, and transformed the insert into competent MGAS2221 cells. Following electroporation, transformants were selected on THY agar plates containing erythromycin and verified by PCR and targeted sequencing. The created strain, M1Δ*hsdR*, was plasmid-complemented by transforming with plasmid pHsdR, which we created by PCR-amplifying the wild-type *hsdR* gene from MGAS2221 and inserting it into the shuttle vector pDCBB (66). For use in conjugation assays, a kanamycin-resistant derivative of M1Δ*hsdR* was created via the same method described in the preceding section (creating strain M1^KAN^Δ*hsdR*).

### RNA-Seq analysis of GAS exposed to human plasma

We wished to determine how the presence/absence of RD2 influenced GAS gene expression upon exposure to human plasma. Total RNA was isolated from the parental M28 isolate MGAS6180 and its RD2 deletion mutant derivative M28ΔRD2. To do this, triplicate cultures of these strains were grown to the exponential phase of growth (O.D._600_ = 0.5) in THY broth, and the bacteria from 8 mL of each culture were pelleted by centrifugation, resuspended in 10 mL of pre-warmed (37°C) human plasma, and returned to the 37°C incubator. Samples were removed after 15 minutes (6 mL) and 60 minutes (4 mL), with GAS being pelleted by centrifugation (5,000 *g* for 5 minutes at room temperature) and resuspended in 6 mL of a solution consisting of one part PBS and two parts RNAprotect bacteria reagent (Qiagen Inc). Following a 5-min incubation at room temperature, GAS were again pelleted by centrifugation (5,000 *g* for 10 minutes at 4°C), the supernatant was discarded, the cell pellets snap-frozen in liquid nitrogen, and the frozen pellets placed at −80°C until ready for processing. Cells were processed using a mechanical lysis method with lysing matrix B tubes in conjunction with a FastPrep24 homogenizer (MP Biomedicals). RNA was isolated using the miRNeasy kit (Qiagen) with contaminating DNA being removed with three treatments with TURBO-DNase-free (Life Technologies). The quality and quantity of the purified RNA were determined using a Bioanalyzer system (Agilent Tech).

Ribosomal RNAs were depleted from the isolated total RNA samples using the RiboMinus Bacteria Transcriptome Isolation Kit (Invitrogen). The rRNA-depleted RNA was then used to generate cDNA libraries for sequencing using the ScriptSeq V2 kit (Illumina, Inc.). Briefly, RNA was fragmented, cDNA was synthesized using random hexamers containing a 5′ tagging sequence, RNA was hydrolyzed, and the cDNA was tagged at the 3′ end. A limited number of PCR cycles (*n* = 14) were used to amplify the libraries via the 5′ and 3′ tags (the libraries were barcoded using different primers), and the libraries were size-selected (170 to 300 bp). The size-selected and barcoded libraries were run on an Illumina flow cell using an Illumina HiSeq4000 instrument. Data were analyzed using CLC Genomics Workbench and normalized to the overall sequencing depth using total mapped read data. Statistical significance was tested using Kal’s Z test with a false-discovery rate correction. The RNAseq data have been deposited at the Gene Expression Omnibus (GEO) database at the National Center for Biotechnology Information (http://www.ncbi.nlm.nih.gov/geo) and are accessible through accession number GSE138408.

### Taqman-based quantitative RT-PCR analysis

To verify the RNA-Seq data, we performed Taqman-based qRT-PCR analyses. Cultures of MGAS6180, M28ΔRD2, and the complemented derivative M28.RD2^COMP^ (Table 1) were processed in an identical manner as they were for the RNA-Seq analysis. Following isolation of total RNA samples, they were converted into cDNA using the reverse transcriptase Superscript III (Invitrogen). The generated cDNA was analyzed via TaqMan-based qRT-PCR analysis using a CFX Connect Real-Time System (Bio-Rad). TaqMan primers and probes for genes of interest, and the internal control gene *proS*, are shown in Table S1. Transcript levels were determined using the ΔΔCT method.

## SUPPLEMENTAL MATERIAL

Supplemental material iai.00273-24-s0001.pdf Fig. S1; Tables S1 to S3.

ASM does not own the copyrights to Supplemental Material that may be linked to, or accessed through, an article. The authors have granted ASM a non-exclusive, world-wide license to publish the Supplemental Material files. Please contact the corresponding author directly for reuse.
