# Supplementary material for: The group A Streptococcus pathogenicity island RD2: virulence role and barriers to conjugative transfer
Source: Infect Immun. 2024 Nov 27;93(1):e00273-24. doi: 10.1128/iai.00273-24 (PMC11784354; doi:10.1128/iai.00273-24)
Supplement: Supplemental material — Fig. S1; Tables S1 to S3. [file iai.00273-24-s0001.pdf]

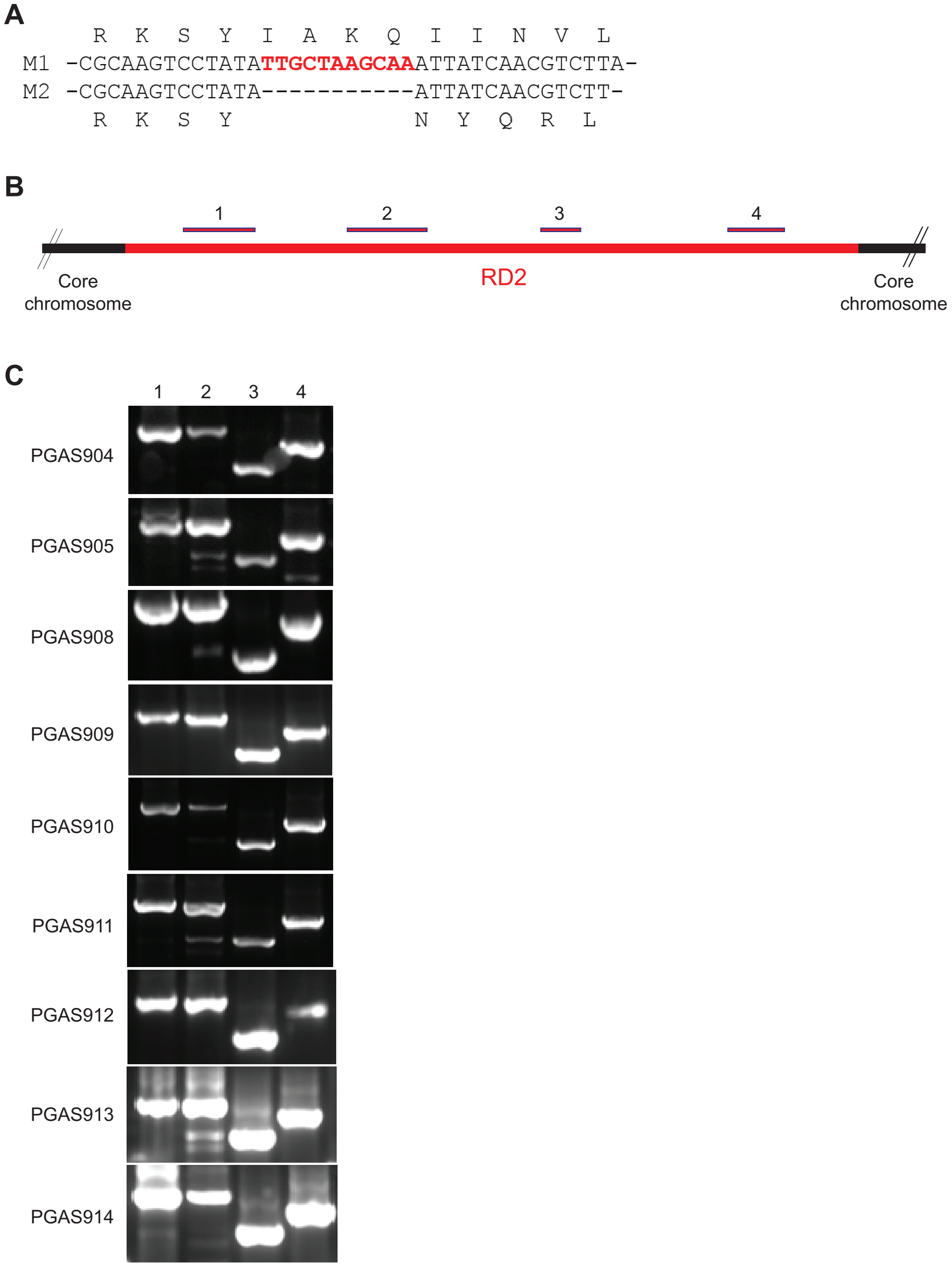

Figure S1

The absence of capsule is not a prerequisite for harboring RD2. (**A**) Comparison of partial *hasB* sequences from M1 GAS and the M2 GAS isolate MGAS10270. Note the frameshifting deletion present in the M2 isolate (highlighted by the red nucleotides in the M1 sequence). (**B**) Schematic showing the relative locations of the four regions of RD2 that were tested in a PCR-base verification of the presence of RD2. RD2 is shown in red, while adjacent regions are shown in black. (**C**) PCR products of the expected size were generated using genomic DNAs from the indicated serotype M2 strains, consistent with these strains harboring RD2.

Primer/probe Name	Sequence (5' to 3')	Function
UNR806	GGTCAGTTTGAATTTGTGAG	To use with UNR805 to check presence and absence of RD2 (filtermating assay)
UNR805	CCCAAATATCTACTCCGTT	To use with UNR805 to check presence and absence of RD2 (filtermating assay)
UNR286	CTTCCTCTTTCTGAAATCATCTTTACC	To use with UNR306 to check transposon adjacent to RD2 in donor strain (filter matingassay)
UNR306	CAGCGGCTACCTTCTTATAATAC	To use with UNR286 to check transposon adjacent to RD2 in donor strain (filter matingassay)
PCR1F	GAATCTGAGTTTTAACCCAGTCTTC	PCR tiling of RD2 (notethat this primer flanks RD2)
PCR1R	CAGGTGCTAGACATACTTATGG	PCR tiling of RD2
PCR2F	CCCAAATATCTACTCCGTT	PCR tiling of RD2
PCR2R	GGTCAGTTTGAATTTGTGAG	PCR tiling of RD2
PCR3F	GGATCTGCTACATCATTAGC	PCR tiling of RD2
PCR3R	CATGCTAAGGATAATCTTAGTGCCA	PCR tiling of RD2
PCR4F	GATTATCGAAAACACCTTGTTCTGC	PCR tiling of RD2
PCR4R	GGATAAAGAAGATGCCTCGTCTAC	PCR tiling of RD2
PCR5F	CTGATGACTGCATCAGAATCC	PCR tiling of RD2
PCR5R	CTGGTTAGTGTAGAACAAGAAGAGG	PCR tiling of RD2
PCR6F	CTTGCTTCGGTAAACTCTTGAC	PCR tiling of RD2
PCR6R	ATCCGATTATCTCAATCTAAGGC	PCR tiling of RD2
PCR7F	GAATCAACCAGCGAATAGTACGTTC	PCR tiling of RD2
PCR7R	CACGTGGACTTGTTGTTGGTATTTTC	PCR tiling of RD2
PCR8F	CAGAGCGTTTAATTTTGGAAGC	PCR tiling of RD2
PCR8R	TCAATGCTCGCTTGATGAAT	PCR tiling of RD2
PCR9F	GACACGATACTTTCAAGTAAAG	PCR tiling of RD2
PCR9R	AGGGTTGGATATGAGTACAGCAGT	PCR tiling of RD2
PCR10F	CTGTACCATCAGAATATTCAATACGTG	PCR tiling of RD2
PCR10R	CAACTCCATTCTTTAATGTTGCAGC	PCR tiling of RD2
PCR11F	GGTGAAAAGTTTAGTGACATTTTAGG	PCR tiling of RD2
PCR11R	GCCAGAGGCTAAGCATAGGA	PCR tiling of RD2
PCR12F	ATACGTTGAATTTGATGGCAA	PCR tiling of RD2
PCR12R	GGTTTCGAGATGTCTTTTTAAAGAATC	PCR tiling of RD2
PCR13F	CGTAGCAATGCTATTGATCCTTTAC	PCR tiling of RD2
PCR13R	ATGTTGCGGGCACCAACCTGAATG	PCR tiling of RD2 (notethat this primer flanks RD2)
UNR1139	AACTACCTAAGGGCGAATTCTGCAGATGatatgatcaaacatcctgctag	Use with HasAD to amplify one flank of *hasA* for use in creating GAS strain $M1^{Kan}\Delta$hasA
UNR1138	actagtaacggccgccagtgtCCTTTAAATGGCTCATAAAGGAAAG	Use with UNR1099 to amplify one flank of *hasA* for use in creating GAS strain $M1^{Kan}\Delta$hasA
UNR1099	CATTGCAAGCATATCTATCTTG	Use with UNR1138 to amplify one flank of *hasA* for use in creating GAS strain $M1^{Kan}\Delta$hasA
HasAB	CGTTACGTTATTAGTTATAGTTATTATAACATGTATTTAAAAAAAGATTTATTCCATCGATTTTGTTGC	Use with UNR1139 to amplify one flank of *hasA* for use in creating GAS strain $M1^{Kan}\Delta$hasA
UNR1137	CTTTCCTTTATGAGCCATTTAAAGGacactggcggccgttactagt	Use with UNR1140 to amplify the erythromycin resistance cassette from GAS strain PGAS490
UNR1140	ctagcaggatgtttgatcatatCATCTGCAGAATTCGCCCTTAGGTAGTT	Use with UNR1137 to amplify the erythromycin resistance cassette from GAS strain PGAS490
UNR1016	CGTTATTAGTTATAGTTATTATAACATGTATTAATCAAATAGGCTTTCAAGTGTGTC	Use with UNR1017 to amplify a a flanking region of the putative transposase gene M28_Spy1335 to help create strain $M28.RD2^{SPEC2}$
UNR1017	TTTCAATATCTCTATCACTTAACTTACC	Use with UNR1016 to amplify a a flanking region of the putative transposase gene M28_Spy1335 to help create strain $M28.RD2^{SPEC2}$
UNR1019	CTATTTAAATAACAGATTAAAAAAATTATAACAATCCCAGCTTTGCCAGTGTGTTTACA C	Use with UNR1021 to amplify a a flanking region of the putative transposase gene M28_Spy1335 to help create strain $M28.RD2^{SPEC2}$
UNR1021	AATGATCCAGCAGGTAAAAATCAGCAAGTC	Use with UNR1019 to amplify a a flanking region of the putative transposase gene M28_Spy1335 to help create strain $M28.RD2^{SPEC2}$
UNR1015	GACACACTTGAAAGCCTATTTGATTAATACATGTTATAATAACTATAACTAATAACG	Use with UNR1020 to amplify the spectinomycin resistance cassette from pSL60 for use in creating strain $M28.RD2^{SPEC2}$
UNR1020	GTGTAAACACACTGGCAAAGCTGGGATTGTTATAATTTTTTTAATCTGTTATTTAAATA G	Use with UNR1015 to amplify the spectinomycin resistance cassette from pSL60 for use in creating strain $M28.RD2^{SPEC2}$
UNR1127	attgctagcgtacggccgtcgacgAACCGTAGATTCAGATACTTATATC	Use with UNR1128 to amplify *hasAB* from MGAS2221 for use in creating GAS strain $M28.hasAB^{FIX}$
UNR1128	caaattttcatcaagctctagttcGCTTGGAGGTTGT ATTCTAATTCAAAG	Use with UNR1127 to amplify *hasAB* from MGAS2221 for use in creating GAS strain $M28.hasAB^{FIX}$
UNR1129	CTTTGAATTAGAATACAACCTCCAAGCgaactagagcttgatgaaaatttg	Use with UNR1130 to amplify pBBL740 for use in creating GAS strain $M28.hasAB^{FIX}$
UNR1130	GATATAAGTATCTGAATCTACGGTTcgtcgacggccgtacgctagcaat	Use with UNR1129 to amplify pBBL740 for use in creating GAS strain $M28.hasAB^{FIX}$
SRNA1E	GTATGAACGTATTTATAAAGACC	Use with SRNA1F to amplify the kanamycin resistance gene from strain $M1^{KAN}$ for use in making strains $M28^{KAN}\Delta RD2.hasAB^{FIX}$ and $M28^{KAN}\Delta RD2$.
SRNA1F	GAGATTCTATTGCCAAAATGG	Use with SRNA1E to amplify the kanamycin resistance gene from strain $M1^{KAN}$ for use in making strains $M28^{KAN}\Delta RD2.hasAB^{FIX}$ and $M28^{KAN}\Delta RD2$.
PROSTMF	TACCACTGGCAAATCGTACC	Taqman primer for *proS*
PROSTMR	CATTTCAACAGCACCGATCT	Taqman primer for *proS*
PROSTMP	CACGCATGATGGTCTTGAATTTCTCA	Taqman probe for *proS*
SKATMF	CGGCTACTTTGAGGTCATTGATT	Taqman primer for *ska*
SKATMR	CCGAATCATCTCGGTCAGCAA	Taqman primer for *ska*
SKATMP	CAAGCGATGCAACCATTACTGATCGAAAC	Taqman probe for *ska*
MRPTMF	TGGCAATAAAGAGGTTCCAACA	Taqman primer for *mrp*
MRPTMR	TGCAGTGAAGAATGGGTTGGT	Taqman primer for *mrp*
MRPTMP	AAACAAAGAGACAATTACCGTCAACAGGCG	Taqman probe for *mrp*
ENDOSTMF	CGCAAGGACGAGGACAAAGC	Taqman primer for *endoS*
ENDOSTMR	CCCACCTGTCTTAGGTTGCCA	Taqman primer for *endoS*
ENDOSTMP	TGCATACCGTTCGGCACGCGTTCCAGT	Taqman probe for *endoS*
UNR866	GCAAAGTTACTTGCAATTTGATCAG	Use with UNR868 to amplify a flank next to hsdR for use in making an *hsdR* mutant derivative of MGAS2221
UNR867	ATGGGAAATCAAACTAAAACGGAACTTGATCACTCATCATGTTCATATTTATCAG	Use with UNR871 to amplify the ErmR gene for use in making a *hsdR* mutant of MGAS2221
UNR868	CTGATAAATATGAACATGATGAGTGATCAAGTTCCGTTTTAGTTTGATTTCCCAT	Use with UNR866 to amplify a flank next to hsdR for use in making an *hsdR* mutant derivative of MGAS2221
UNR869	GGCTGTTTAGATTTTGTCATCTC	Use with UNR872 to amplify a flank next to hsdR for use in making an *hsdR* mutant derivative of MGAS2221
UNR871	CTACTTTCAAAGATACGGTTGGTTCGTGCCTTAGGTAGTTTATGCATCCCT	Use with UNR867 to amplify the ErmR gene for use in making a *hsdR* mutant of MGAS2221
UNR872	AGGGATGCATAAACTACCTAAGGCACGAACCAACCGTATCTTTGAAAGTAG	Use with UNR869 to amplify a flank next to hsdR for use in making an *hsdR* mutant derivative of MGAS2221
UNR953	CACGATTGGCTTAATCGCCTTAAGTTCTGAgactgtttggaaggtttcaatagcttcg	Use with UNR869 to amplify *hsdR* from MGAS2221 to make the insert for plasmid pHsdR
UNR954	cgaagctattgaaaccttccaaacagtcTCAGAACTTAAGGCGATTAAGCCAATCGTG	Use with UNR955 to amplify pDCBB for use in making the vector backbone of pHsdR
UNR955	GAGATGACAAAATCTAAACAGCcatgcaacctctgtttgtttttcgccg	Use with UNR954 to amplify pDCBB for use in making the vector backbone of pHsdR

Table S1
Primers used in this study.

Gene name	Fold-difference in the RD2 deletion mutant realtive to the parental strain	Left gene border	Right gene border
M28_Spy1632	-5.60	1,635,170	1,635,512
comEA	-4.13	1,148,631	1,149,294
rpsN	-3.46	70,482	70,668
rpmD	-3.36	72,944	73,127
ssb2.1	-3.07	1,260,446	1,260,866
dnaQ	-2.98	1,569,692	1,570,319
mrp	-2.95	1,712,343	1,713,498
rpsS	-2.72	66,846	67,179
rplX	-2.61	69,595	69,901
M28_Spy0851	-2.61	871,994	872,114
radC	-2.61	836,802	837,483
M28_Spy1006	-2.59	1,017,152	1,017,323
M28_Spy0944	-2.58	962,441	962,642
M28_Spy1265	-2.55	1,259,444	1,259,801
M28_Spy1277	-2.53	1,263,753	1,264,098
M28_Spy1261	-2.49	1,258,238	1,258,643
M28_SpyR0017	-2.46	1,605,215	1,608,116
M28_Spy1029	-2.43	1,028,544	1,028,703
M28_Spy0696	-2.41	719,626	719,929
M28_Spy1007	-2.35	1,017,319	1,017,556
M28_Spy1834	-2.33	1,840,396	1,840,552
M28_Spy0209	-2.32	224,226	225,057
prtF1	-2.30	116,836	118,687
M28_SpyR0008	-2.28	81,330	84,231
mutT	-2.28	1,187,237	1,187,693
sagG	-2.27	562,682	563,606
M28_Spy1302	-2.26	1,285,001	1,286,135
sagD	-2.24	559,975	561,334
M28_Spy0733	-2.24	754,738	755,026
M28_Spy0963	-2.24	983,217	983,589
M28_SpyR0011	-2.23	270,513	273,414
serS	-2.23	1,468,176	1,469,454
M28_Spy0450	-2.22	456,406	456,580
M28_Spy0987	-2.22	1,003,661	1,004,015
M28_SpyR0005	-2.20	25,041	27,942
M28_Spy1108	-2.20	1,113,721	1,113,985
M28_Spy0948	-2.17	964,081	964,330
M28_Spy0074	-2.16	86,950	87,178
M28_Spy1259	-2.15	1,257,568	1,258,075
M28_Spy0150	-2.09	168,557	169,421
M28_Spy1031	-2.09	1,030,225	1,031,035
M28_Spy1266	-2.06	1,259,797	1,260,238
M28_Spy1539	-2.06	1,541,222	1,541,879
M28_Spy1714	-2.04	1,724,184	1,724,481
M28_Spy0036	-2.03	55,056	55,494
M28_Spy0262	-2.03	282,182	283,025
M28_Spy0700	-2.02	721,678	722,779
M28_Spy1104	-2.02	1,109,129	1,110,263
rplO	-2.01	73,341	73,782
sagH	-1.99	563,614	564,742
M28_Spy1030	-1.98	1,029,469	1,030,213
M28_Spy1107	-1.98	1,112,859	1,113,669
rplN	-1.97	69,148	69,517

rplR	-1.96	72,051	72,417
sagI	-1.96	564,738	565,857
M28_Spy0073	-1.95	86,602	86,911
rplP	-1.95	68,208	68,622
enn	-1.94	1,709,794	1,710,727
M28_Spy0379	-1.94	395,578	395,812
M28_Spy1028	-1.93	1,027,915	1,028,515
adk	-1.93	75,252	75,891
ntpA	-1.92	144,634	146,410
M28_Spy1417	-1.92	1,414,163	1,414,430
epf	-1.90	550,382	556,703
rplV	-1.90	67,194	67,539
M28_Spy0571	-1.90	588,845	589,514
M28_Spy0771	-1.90	799,441	799,582
M28_Spy0978	-1.89	994,656	996,711
atpE	-1.89	573,520	573,718
M28_Spy1264	-1.89	1,259,196	1,259,448
sclA	-1.88	1,678,739	1,680,107
M28_Spy1863	-1.88	1,855,695	1,856,139
M28_Spy1012	-1.85	1,019,579	1,021,133
M28_Spy0480	-1.85	483,798	484,302
M28_Spy1260	-1.84	1,258,071	1,258,242
M28_Spy0102	-1.84	113,129	114,179
gatC	-1.81	1,491,675	1,491,990
ung	-1.81	708,667	709,321
acoA	-1.80	751,096	752,077
M28_Spy1454	-1.80	1,452,553	1,452,850
M28_Spy1137	-1.79	1,143,969	1,144,218
manM	-1.78	1,465,574	1,466,384
M28_Spy0263	-1.78	283,351	284,197
ssb1	-1.77	1,260,858	1,261,533
M28_Spy1572	-1.77	1,571,062	1,571,554
rplF	-1.77	71,419	71,956
rplW	-1.76	65,613	65,910
M28_Spy0078	-1.76	89,301	89,685
M28_SpyR0002	-1.74	19,040	21,941
M28_Spy0975	-1.74	991,223	993,119
M28_Spy0341	-1.73	362,133	362,643
msrA.2	-1.73	386,087	386,597
M28_Spy0037	-1.72	55,516	55,918
inlA	-1.72	1,105,286	1,107,665
M28_Spy0670	-1.72	690,962	691,133
irr	-1.71	1,719,411	1,720,065
M28_Spy0964	-1.71	983,588	984,287
sagE	-1.69	561,308	561,980
srtR	-1.69	802,079	802,766
M28_Spy1228	-1.69	1,232,807	1,233,104
upp	-1.69	335,472	336,102
M28_Spy1285	-1.69	1,268,008	1,269,163
M28_Spy1067	-1.68	1,071,166	1,071,862
M28_Spy1695	-1.68	1,699,500	1,701,978
M28_Spy1437	-1.68	1,437,747	1,438,365
hsdR	-1.67	1,611,899	1,614,878
M28_Spy0101	-1.67	112,468	113,110
M28_Spy1769	-1.66	1,788,463	1,789,177
M28_Spy1573	-1.65	1,571,550	1,571,790

rplJ	-1.65	798,155	798,656
M28_Spy0172	-1.65	189,927	190,113
sagA	-1.64	557,567	557,729
recO	-1.64	33,469	34,225
M28_Spy1559	-1.64	1,559,460	1,560,213
M28_Spy0940	-1.64	960,453	960,996
M28_Spy1282	-1.63	1,266,604	1,266,964
rpsE	-1.63	72,435	72,930
clpE	-1.63	1,184,827	1,187,110
proC	-1.62	108,068	108,839
M28_Spy1637	-1.62	1,637,434	1,638,166
M28_Spy1500	-1.62	1,492,971	1,493,526
M28_Spy0454	-1.62	459,812	461,180
M28_Spy0756	-1.62	783,220	784,240
M28_Spy1343	-1.62	1,325,961	1,326,717
M28_Spy1698	-1.62	1,703,088	1,704,222
M28_Spy0369	-1.61	386,587	386,809
pstC	-1.61	947,161	948,097
rofA	-1.61	115,112	116,606
M28_Spy1300	-1.60	1,284,578	1,284,917
M28_Spy1233	-1.60	1,236,384	1,237,599
M28_Spy1147	-1.60	1,151,418	1,153,419
M28_Spy1677	-1.60	1,681,364	1,682,183
ccdA	-1.59	1,271,058	1,271,769
M28_Spy0215	-1.59	229,750	230,341
oadA2	-1.59	885,377	886,760
M28_Spy1433	-1.59	1,434,342	1,435,065
M28_Spy1506	-1.59	1,499,466	1,500,171
M28_Spy1086	-1.58	1,089,644	1,089,935
M28_Spy0386	-1.57	398,182	398,479
mreA	-1.57	951,930	952,863
M28_Spy0493	-1.56	497,747	499,049
rpsL	-1.56	240,046	240,460
M28_Spy0952	-1.56	968,981	970,217
spn	-1.56	154,943	156,308
comR	-1.56	52,783	53,695
M28_Spy0904	-1.56	923,636	924,635
clpP	-1.56	336,325	336,916
rplC	-1.55	64,340	64,967
cinA	-1.55	1,801,505	1,802,777
rpsQ	-1.55	68,863	69,124
M28_Spy1791	-1.55	1,809,726	1,810,494
pyrD	-1.55	1,165,325	1,166,273
trmU	-1.54	1,866,295	1,867,417
deoD2	-1.54	700,331	701,045
M28_Spy0237	-1.54	252,771	253,542
M28_Spy0003	-1.53	2,952	3,150
argR2	-1.53	1,824,573	1,825,011
M28_Spy1682	-1.53	1,687,251	1,689,009
rpmC	-1.53	68,631	68,838
pyrF	-1.53	703,844	704,537
M28_Spy1584	-1.53	1,581,462	1,581,888
M28_Spy1360	-1.52	1,351,138	1,352,494
trmD	-1.52	659,798	660,530
M28_Spy0561	-1.52	580,227	580,452
rplE	-1.52	69,924	70,467

citD	-1.52	894,449	894,758
M28_Spy1089	-1.52	1,091,166	1,091,703
M28_Spy1782	-1.51	1,799,591	1,799,990
speC	-1.51	987,710	988,418
rplB	-1.51	65,927	66,761
M28_Spy0813	-1.51	833,101	834,670
braB	-1.51	286,257	287,628
M28_Spy0699	-1.51	721,425	721,812
M28_Spy1666	-1.51	1,665,845	1,666,220
M28_Spy1154	-1.50	1,161,418	1,161,610
murB	-1.50	821,336	822,224
sagF	-1.50	561,976	562,660
M28_Spy0177	-1.50	193,241	193,757
M28_Spy1352	1.50	1,339,092	1,340,016
M28_Spy1616	1.50	1,618,808	1,620,371
ftsW	1.50	490,112	491,387
M28_Spy0912	1.50	931,785	932,850
M28_Spy1050	1.50	1,052,545	1,053,529
M28_Spy1404	1.51	1,402,051	1,402,855
bacA	1.51	249,727	250,567
M28_Spy0207	1.51	221,904	223,224
M28_Spy1393	1.51	1,390,210	1,390,537
M28_Spy0854	1.51	873,633	874,284
M28_Spy1490	1.51	1,485,702	1,486,272
M28_Spy1412	1.51	1,409,393	1,409,615
M28_Spy0833	1.51	852,297	853,581
spd	1.52	1,735,701	1,736,517
M28_Spy1365	1.52	1,360,900	1,361,125
rpoD	1.52	601,242	602,352
M28_Spy1598	1.52	1,595,227	1,596,154
nrdF.1	1.52	354,636	355,650
cfa	1.52	970,586	971,360
M28_Spy1201	1.52	1,208,071	1,208,716
M28_Spy0475	1.52	479,250	480,153
M28_Spy0844	1.53	861,364	863,083
recG	1.53	1,501,378	1,503,394
M28_Spy0729	1.54	748,915	750,823
M28_SpyR0016	1.54	1,605,013	1,605,127
ecsA	1.54	1,457,519	1,458,245
pfl	1.54	1,556,018	1,558,346
M28_Spy1064	1.55	1,066,338	1,068,333
M28_Spy1206	1.55	1,212,036	1,212,576
M28_Spy1517	1.55	1,510,682	1,511,705
M28_Spy1162	1.55	1,168,788	1,169,064
cbiO1	1.55	1,872,299	1,873,190
M28_Spy1052	1.55	1,054,802	1,055,042
M28_Spy0400	1.55	408,021	408,168
M28_SpyR0003	1.56	22,029	22,143
M28_Spy1457	1.56	1,454,992	1,455,628
udk	1.56	1,114,064	1,114,691
hasB	1.57	1,879,042	1,880,251
ptsB	1.57	784,890	785,382
rpsF	1.57	1,542,928	1,543,219
fabH	1.57	1,478,037	1,479,012
cdsA	1.58	1,663,589	1,664,384
M28_Spy1744	1.58	1,753,722	1,755,261
arcB	1.58	1,217,984	1,218,998
nrdH	1.58	1,121,325	1,121,577

M28_Spy1430	1.58	1,430,283	1,431,123
uviB	1.59	1,827,062	1,827,389
M28_Spy0141	1.59	159,493	159,706
M28_Spy0165	1.59	184,193	184,490
M28_Spy1214	1.59	1,221,074	1,221,755
M28_Spy0855	1.59	874,280	874,721
malA	1.59	1,043,571	1,044,387
M28_Spy0501	1.60	507,696	508,896
pgdA	1.60	299,700	301,011
M28_Spy1771	1.60	1,789,979	1,790,837
fasA	1.60	217,070	217,811
pflC	1.60	325,408	326,272
M28_Spy0377	1.60	394,144	394,621
M28_Spy0819	1.60	839,085	839,433
pyk	1.60	977,264	978,767
rbfA	1.61	1,448,821	1,449,178
greA	1.61	308,815	309,298
nagB	1.62	1,140,107	1,140,812
M28_Spy1776	1.62	1,793,879	1,794,026
mraW	1.62	1,408,149	1,409,163
hlyX	1.62	324,029	325,403
ciaH	1.62	938,907	940,218
malG	1.63	1,041,664	1,042,501
rpmH	1.63	220,743	220,878
M28_Spy0146	1.63	165,391	166,825
ptsC	1.64	785,398	786,208
srtG	1.64	811,972	812,695
flaR	1.64	1,691,902	1,692,409
M28_Spy0463	1.64	470,128	470,566
pta	1.64	844,720	845,716
M28_Spy1224	1.65	1,229,794	1,230,661
M28_Spy0014	1.66	15,041	16,502
perR	1.66	180,334	180,802
hrcA	1.66	1,484,533	1,485,568
M28_Spy0414	1.66	420,269	421,604
M28_Spy1348	1.67	1,334,618	1,335,224
accD	1.67	1,469,686	1,470,457
M28_Spy0191	1.67	206,220	208,008
M28_Spy1163	1.67	1,169,162	1,169,750
M28_Spy1366	1.69	1,361,718	1,362,267
lrp	1.69	1,673,202	1,674,093
rocA	1.69	1,349,118	1,350,474
M28_Spy1662	1.69	1,662,257	1,663,517
M28_Spy0550	1.70	569,440	569,941
M28_Spy1362	1.70	1,353,464	1,353,998
fms	1.70	677,282	677,693
glnQ.2	1.70	1,062,626	1,063,367
ahrC.2	1.71	1,221,896	1,222,370
gidB	1.71	291,275	291,989
accB	1.72	1,473,143	1,473,644
nagA	1.72	1,431,244	1,432,393
M28_Spy1896	1.72	1,893,627	1,894,215
M28_Spy1466	1.72	1,461,605	1,463,066
M28_Spy0331	1.73	351,198	351,906
M28_Spy0309	1.74	327,413	328,067
M28_Spy0853	1.75	872,605	873,517
spi_1	1.76	1,551,035	1,551,629
M28_Spy0911	1.77	930,827	931,784
phaB	1.77	1,479,523	1,480,315

M28_Spy0117	1.77	134,304	135,489
sibA	1.77	30,872	32,069
M28_Spy0206	1.77	221,151	221,856
M28_Spy1871	1.79	1,865,202	1,865,520
carB	1.79	643,743	646,920
M28_Spy1429	1.80	1,429,618	1,430,152
M28_Spy0004	1.81	3,479	4,595
M28_Spytm01	1.83	985,914	986,259
M28_Spy1464	1.83	1,460,491	1,461,016
M28_Spy0404	1.84	411,352	412,072
rpsN2	1.84	1,580,004	1,580,274
plr	1.85	243,716	244,754
M28_Spy1897	1.85	1,894,797	1,895,277
M28_Spy1520	1.85	1,513,646	1,517,474
M28_Spy1146	1.85	1,151,116	1,151,395
M28_Spy0252	1.86	274,925	275,510
M28_Spy1778	1.86	1,796,502	1,798,062
nusB	1.87	1,533,899	1,534,352
glpF	1.88	1,420,539	1,421,241
M28_Spy1576	1.89	1,574,258	1,574,534
M28_Spy0487	1.89	491,689	492,937
parB	1.90	1,896,770	1,897,577
fabD	1.90	1,475,616	1,476,561
slaA	1.91	1,227,660	1,228,236
M28_Spy0966	1.93	985,208	985,823
M28_Spy0497	1.93	503,570	505,478
M28_Spy0324	1.93	341,183	341,576
adhA	1.94	60,892	61,909
sptR_2	1.95	681,337	682,570
M28_Spy1889	1.95	1,883,232	1,884,096
M28_Spy1515	1.95	1,508,089	1,509,853
M28_Spy1349	1.97	1,335,529	1,336,975
M28_Spy1161	1.97	1,166,572	1,168,435
secE	1.98	1,747,061	1,747,238
M28_Spy1775	2.00	1,792,915	1,793,851
phnA	2.00	973,968	974,304
scrR	2.01	1,532,793	1,533,759
rpmG	2.01	1,833,946	1,834,096
acpP.2	2.02	1,477,752	1,477,977
kgdA	2.02	512,162	512,798
M28_Spy1717	2.02	1,730,255	1,730,492
M28_Spy1528	2.03	1,529,017	1,529,182
M28_Spy1731	2.03	1,741,925	1,743,230
alr	2.05	1,520,658	1,521,015
M28_Spy0472	2.05	476,998	477,211
M28_Spy0576	2.06	598,623	598,800
M28_Spy1830	2.07	1,837,141	1,837,744
M28_Spy0116	2.08	133,272	134,184
M28_Spy0728	2.08	748,543	748,870
M28_Spy1484	2.09	1,479,012	1,479,447
grpE	2.12	1,483,958	1,484,531
M28_Spy0979	2.15	996,707	997,487
fabK	2.16	1,476,579	1,477,551
M28_Spy1435	2.16	1,436,328	1,436,886
copA	2.17	1,444,936	1,447,168
M28_Spy0580	2.17	602,587	602,926
nrdD	2.18	1,794,207	1,796,406
M28_Spy0956	2.18	973,220	973,862
M28_Spy0300	2.19	319,610	319,874

lacG	2.20	1,625,953	1,627,360
M28_Spy1654	2.20	1,652,603	1,652,873
M28_Spy0416	2.21	424,109	425,102
M28_Spy0499	2.24	506,371	507,154
salB	2.24	1,623,588	1,625,214
nrdG	2.25	1,791,783	1,792,398
mur1.2	2.30	668,358	669,066
fruB	2.33	664,660	665,572
M28_Spy0513	2.33	519,539	520,304
lacD.2	2.34	1,629,470	1,630,454
mac	2.37	670,022	671,048
M28_Spy1575	2.37	1,572,168	1,574,151
M28_Spy0096	2.37	110,063	110,228
M28_Spy1022	2.38	1,025,502	1,025,742
M28_Spy1794	2.39	1,811,108	1,811,516
M28_Spy1805	2.39	1,815,979	1,817,374
ska	2.39	1,674,390	1,675,713
fruA	2.40	665,568	667,515
lacB.2	2.40	1,631,431	1,631,947
clpL	2.47	694,049	696,149
M28_Spy0343	2.48	363,519	364,020
M28_Spy0147	2.56	166,895	167,174
M28_Spy0039	2.56	57,998	60,641
M28_Spy0390	2.60	400,476	400,875
lacA.2	2.61	1,631,981	1,632,410
M28_Spy1812	2.64	1,820,084	1,820,468
czcD	2.64	657,543	658,419
M28_Spy1614	2.65	1,617,880	1,618,072
M28_Spy0301	2.71	320,015	320,600
lacF	2.72	1,629,129	1,629,447
M28_Spy1770	2.74	1,789,455	1,789,695
M28_Spy0330	2.75	350,916	351,096
dnaK	2.76	1,481,951	1,483,778
scrA	2.78	1,529,224	1,531,108
atoE	2.79	131,763	133,203
M28_Spy0530	2.79	539,114	539,330
M28_Spy1232	2.80	1,236,068	1,236,398
M28_Spy0469	2.90	473,984	474,269
endoS	2.90	1,525,979	1,528,994
lacC.2	2.90	1,630,456	1,631,386
M28_Spy1003	2.94	1,015,753	1,016,248
lctO	3.08	344,035	345,223
M28_Spy1193	3.18	1,201,408	1,201,660
M28_Spy1421	3.20	1,419,084	1,420,524
agaD	3.21	505,563	506,385
M28_Spy0333	3.21	354,508	354,640
lacE	3.24	1,627,432	1,629,130
M28_Spy0154	3.26	172,350	173,442
msmK	3.32	1,671,771	1,672,905
agaV	3.33	507,172	507,661
M28_Spy1072	3.33	1,074,369	1,075,176
silD	3.38	398,004	398,133
dnaJ	3.52	1,480,534	1,481,671
M28_Spy0417	3.59	425,103	425,922
fruR	3.73	663,950	664,664
M28_Spy0362	3.73	381,595	381,949
M28_Spy1257	4.00	1,256,320	1,256,542
M28_Spy1351	4.11	1,337,991	1,338,942
M28_Spy0633	4.14	656,952	657,354

M28_Spy1746	4.23	1,755,983	1,756,250
M28_Spy1799	4.69	1,813,290	1,813,860
copZ	6.86	1,444,719	1,444,923
M28_Spy1160	7.25	1,166,327	1,166,501

Table S2

Statistically significant genes (Kal's Z-test with FDR correction) that show a minimum of 1.5-fold difference in mRNA abundances between the parental M28 isolate and its isogenic RD2 deletion mutant derivative following a 15 min exposure to human plasma

Gene name	Fold-difference in the RD2 deletion mutant realtive to the parental strain	Left gene border	Right gene border
M28_Spy0015	1.53	16,843	16,978
M28_SpyR0002	-1.70	19,040	21,941
M28_SpyR0005	-1.69	25,041	27,942
M28_Spy0039	1.84	57,998	60,641
adhA	1.95	60,892	61,909
M28_Spy0072	1.68	85,779	85,965
adcR	-1.60	87,287	87,731
M28_Spy0083	-2.15	101,109	101,475
M28_Spy0096	1.85	110,063	110,228
rofA	-1.58	115,112	116,606
srtB	-1.53	123,925	124,666
atoE	3.03	131,763	133,203
M28_Spy0116	2.31	133,272	134,184
M28_Spy0117	1.55	134,304	135,489
ntpC	-1.99	143,118	144,117
M28_Spy0141	1.90	159,493	159,706
M28_Spy0146	2.66	165,391	166,825
M28_Spy0147	3.03	166,895	167,174
M28_Spy0148	2.65	167,296	167,782
opuABC	-1.67	175,140	176,868
M28_Spy0161	1.57	181,479	182,814
nadC	1.53	186,400	187,300
speG	1.72	196,002	196,707
M28_Spy0184	1.50	198,860	200,369
M28_Spy0190	-1.52	204,514	206,221
M28_Spy0192	1.61	208,125	208,893
M28_Spy0195	-1.55	211,076	211,577
M28_Spy0206	3.57	221,151	221,856
M28_Spy0207	1.75	221,904	223,224
M28_Spy0208	2.59	223,251	224,214
M28_Spy0210	2.49	225,213	225,876
M28_Spy0215	1.70	229,750	230,341
M28_Spy0238	-1.50	253,636	254,899
M28_Spy0250	1.60	268,316	268,451
M28_SpyR0011	-2.16	270,513	273,414
M28_Spy0262	-1.86	282,182	283,025
M28_Spy0263	-1.79	283,351	284,197
lemA	-1.54	292,138	292,696
M28_Spy0272	-1.63	292,742	293,639
M28_Spy0287	-1.51	310,922	311,234
M28_Spy0291	1.84	313,619	313,862
pflC	1.86	325,408	326,272
M28_Spy0326	-2.38	342,550	342,907
lctO	1.62	344,035	345,223
M28_Spy0331	1.84	351,198	351,906
M28_Spy0333	2.02	354,508	354,640
M28_Spy0400	1.81	408,021	408,168
M28_Spy0440	2.10	449,922	451,581
M28_Spy0448	1.68	455,153	455,885
licT	1.81	461,507	462,350
M28_Spy0459	-1.55	466,570	467,473
M28_Spy0481	-1.74	484,285	484,669
M28_Spy0501	1.77	507,696	508,896
rplS	1.89	538,406	538,754

M28_Spy0530	1.84	539,114	539,330
M28_Spy0531	1.60	539,767	540,337
epf	-1.75	550,382	556,703
sagA	-1.51	557,567	557,729
sagF	-1.52	561,976	562,660
carB	2.07	643,743	646,920
czcD	2.30	657,543	658,419
fruR	2.81	663,950	664,664
fruB	2.68	664,660	665,572
fruA	1.81	665,568	667,515
mac	1.72	670,022	671,048
M28_Spy0650	2.06	671,551	671,728
clpL	1.58	694,049	696,149
M28_Spy0694	1.54	717,254	717,485
M28_Spy0696	-1.85	719,626	719,929
M28_Spy0698	-2.20	721,185	721,377
acoA	-1.58	751,096	752,077
acoB	-1.75	752,132	753,134
acoC	-1.96	753,318	754,728
M28_Spy0733	-1.78	754,738	755,026
msrB	-1.58	782,653	783,091
M28_Spy0757	2.00	784,446	784,872
ptsB	1.63	784,890	785,382
M28_Spy0771	2.10	799,441	799,582
M28_Spy0795	-1.58	816,323	817,286
guaC	-1.74	850,428	851,412
M28_Spy0851	-1.97	871,994	872,114
M28_Spy0874	3.23	892,320	892,644
citE	-1.54	894,745	895,633
citX	-1.72	897,160	897,739
M28_Spy0895	1.52	913,747	914,614
rnhB	-1.51	916,379	916,736
M28_Spy0903	-1.54	923,262	923,595
mreA	-1.56	951,930	952,863
truB	-1.53	952,891	953,776
M28_Spy0942	-1.54	961,138	961,780
M28_Spy0943	-1.61	961,942	962,431
M28_Spy0944	-1.57	962,441	962,642
M28_Spy0947	-1.52	963,433	964,045
M28_Spy0948	-1.75	964,081	964,330
M28_Spy0967	2.67	986,413	986,602
M28_Spy1021	1.78	1,025,150	1,025,357
M28_Spy1055	2.01	1,057,862	1,058,006
glnH	2.91	1,060,452	1,062,627
glnQ.2	2.01	1,062,626	1,063,367
M28_Spy1062	1.54	1,065,666	1,066,002
M28_Spy1064	1.51	1,066,338	1,068,333
M28_Spy1067	1.54	1,071,166	1,071,862
M28_Spy1070	2.69	1,073,463	1,073,592
M28_Spy1071	-3.18	1,073,833	1,074,349
M28_Spy1072	4.62	1,074,369	1,075,176
M28_Spy1104	-1.64	1,109,129	1,110,263
M28_Spy1108	1.94	1,113,721	1,113,985
M28_Spy1146	1.77	1,151,116	1,151,395
M28_Spy1160	2.44	1,166,327	1,166,501
M28_Spy1161	1.81	1,166,572	1,168,435
M28_Spy1197	2.11	1,205,180	1,205,384
M28_Spy1214	1.92	1,221,074	1,221,755

M28_Spy1247	1.87	1,248,678	1,248,918
M28_Spy1259	-1.96	1,257,568	1,258,075
M28_Spy1260	-1.98	1,258,071	1,258,242
M28_Spy1261	-1.89	1,258,238	1,258,643
M28_Spy1263	-1.60	1,258,918	1,259,203
M28_Spy1264	-2.02	1,259,196	1,259,448
M28_Spy1265	-2.47	1,259,444	1,259,801
M28_Spy1266	-1.70	1,259,797	1,260,238
M28_Spy1267	-2.21	1,260,237	1,260,441
ssb2.1	-2.08	1,260,446	1,260,866
ssb1	-3.44	1,260,858	1,261,533
M28_Spy1271	-2.25	1,262,037	1,262,313
M28_Spy1274	-3.30	1,262,653	1,263,067
M28_Spy1275	-3.92	1,263,188	1,263,446
M28_Spy1277	-3.34	1,263,753	1,264,098
M28_Spy1278	-2.71	1,264,256	1,264,511
M28_Spy1280	-2.17	1,264,878	1,265,607
M28_Spy1281	-2.42	1,265,617	1,265,809
M28_Spy1302	-1.55	1,285,001	1,286,135
M28_Spy1350	1.97	1,337,055	1,337,982
M28_SpyR0013	1.56	1,355,733	1,355,847
M28_Spy1365	2.89	1,360,900	1,361,125
M28_Spy1381	1.52	1,377,778	1,378,096
gmk	1.58	1,378,111	1,378,747
M28_Spy1420	2.00	1,417,595	1,419,095
M28_Spy1421	2.59	1,419,084	1,420,524
glpK	-1.79	1,423,096	1,424,623
M28_Spy1425	-1.64	1,424,982	1,425,435
M28_Spy1434	-1.51	1,435,193	1,436,036
M28_Spy1443	2.57	1,442,737	1,443,043
M28_Spy1444	1.83	1,443,035	1,443,509
copZ	1.89	1,444,719	1,444,923
copA	2.01	1,444,936	1,447,168
copY	1.64	1,447,167	1,447,605
M28_Spy1465	1.67	1,460,990	1,461,452
accD	1.56	1,469,686	1,470,457
fabZ	1.84	1,472,724	1,473,147
accB	1.76	1,473,143	1,473,644
fabF	1.61	1,473,645	1,474,878
M28_Spy1479	1.51	1,474,892	1,475,627
fabD	1.73	1,475,616	1,476,561
fabK	1.66	1,476,579	1,477,551
dnaJ	1.55	1,480,534	1,481,671
dnaK	1.64	1,481,951	1,483,778
grpE	1.73	1,483,958	1,484,531
hrcA	1.87	1,484,533	1,485,568
M28_Spy1493	1.65	1,488,100	1,488,340
endoS	2.63	1,525,979	1,528,994
M28_Spy1528	2.37	1,529,017	1,529,182
scrA	1.82	1,529,224	1,531,108
M28_Spy1573	1.51	1,571,550	1,571,790
M28_Spy1575	1.70	1,572,168	1,574,151
M28_Spy1576	3.59	1,574,258	1,574,534
glnA	1.76	1,585,013	1,586,360
M28_Spy1589	1.74	1,586,397	1,586,769
M28_Spy1602	1.52	1,600,573	1,601,419
M28_Spy1604	1.66	1,602,057	1,602,528
M28_SpyR0017	-1.55	1,605,215	1,608,116

M28_Spy1614	1.61	1,617,880	1,618,072
M28_Spy1613	2.82	1,618,046	1,618,166
M28_Spy1616	1.55	1,618,808	1,620,371
M28_Spy1618	1.55	1,622,319	1,623,057
salB	1.94	1,623,588	1,625,214
lacE	1.84	1,627,432	1,629,130
lacF	2.77	1,629,129	1,629,447
lacD.2	2.17	1,629,470	1,630,454
lacC.2	2.02	1,630,456	1,631,386
lacB.2	2.80	1,631,431	1,631,947
lacA.2	1.77	1,631,981	1,632,410
M28_Spy1636	1.97	1,637,231	1,637,438
M28_Spy1652	1.68	1,650,190	1,652,353
M28_Spy1666	-1.74	1,665,845	1,666,220
msmK	1.54	1,671,771	1,672,905
ska	3.28	1,674,390	1,675,713
flaR	1.64	1,691,902	1,692,409
M28_Spy1698	-1.89	1,703,088	1,704,222
M28_Spy1702	1.97	1,710,937	1,712,119
mrp	-4.38	1,712,343	1,713,498
M28_Spy1714	-1.50	1,724,184	1,724,481
M28_Spy1727	-1.57	1,736,880	1,737,390
M28_Spy1731	2.49	1,741,925	1,743,230
M28_Spy1732	3.23	1,743,239	1,743,569
M28_Spy1733	3.14	1,743,575	1,743,896
M28_Spy1736	2.12	1,746,051	1,746,825
M28_Spy1746	2.13	1,755,983	1,756,250
M28_Spy1763	1.51	1,776,944	1,780,019
M28_Spy1771	1.53	1,789,979	1,790,837
M28_Spy1776	1.64	1,793,879	1,794,026
nrdD	1.92	1,794,207	1,796,406
M28_Spy1778	1.67	1,796,502	1,798,062
tag	-3.06	1,802,845	1,803,406
ruvA	-2.77	1,803,415	1,804,012
lmrP	-2.16	1,804,013	1,805,234
mutL	-2.20	1,805,244	1,807,227
M28_Spy1789	-1.65	1,807,321	1,808,485
M28_Spy1790	2.84	1,808,721	1,808,865
M28_Spy1794	2.07	1,811,108	1,811,516
M28_Spy1799	2.08	1,813,290	1,813,860
M28_Spy1803	2.69	1,814,828	1,815,101
M28_Spy1809	-1.51	1,818,272	1,818,782
M28_Spy1811	3.38	1,819,747	1,820,110
argR2	1.69	1,824,573	1,825,011
rpmG	2.39	1,833,946	1,834,096
M28_Spy1871	1.52	1,865,202	1,865,520
M28_Spy1887	1.58	1,881,654	1,882,068
M28_Spy1897	1.77	1,894,797	1,895,277
htrA	-1.51	1,895,488	1,896,712

Table S3

Statistically significant genes (Kal's Z-test with FDR correction) that show a minimum of 1.5-fold difference in mRNA abundances between the parental M28 isolate and its isogenic RD2 deletion mutant derivative following a 60 min exposure to human plasma
